# Architecting the metabolic reprogramming survival risk framework in LUAD through single-cell landscape analysis: three-stage ensemble learning with genetic algorithm optimization

**DOI:** 10.1186/s12967-024-05138-2

**Published:** 2024-04-15

**Authors:** Xinti Sun, Minyu Nong, Fei Meng, Xiaojuan Sun, Lihe Jiang, Zihao Li, Peng Zhang

**Affiliations:** 1 Department of Cardiothoracic Surgery, Tianjin Medical University General Hospital, Tianjin, China; 2 School of Clinical Medicine, Youjiang Medical University for Nationalities, Baise, Guangxi China; 3 Department of Oncology, Qingdao University Affiliated Hospital, Qingdao, Shandong China

**Keywords:** Ensemble learning, Genetic algorithm, Metabolic reprogramming, Web tool

## Abstract

Recent studies have increasingly revealed the connection between metabolic reprogramming and tumor progression. However, the specific impact of metabolic reprogramming on inter-patient heterogeneity and prognosis in lung adenocarcinoma (LUAD) still requires further exploration. Here, we introduced a cellular hierarchy framework according to a malignant and metabolic gene set, named malignant & metabolism reprogramming (MMR), to reanalyze 178,739 single-cell reference profiles. Furthermore, we proposed a three-stage ensemble learning pipeline, aided by genetic algorithm (GA), for survival prediction across 9 LUAD cohorts (*n* = 2066). Throughout the pipeline of developing the three stage-MMR (3 S-MMR) score, double training sets were implemented to avoid over-fitting; the gene-pairing method was utilized to remove batch effect; GA was harnessed to pinpoint the optimal basic learner combination. The novel 3 S-MMR score reflects various aspects of LUAD biology, provides new insights into precision medicine for patients, and may serve as a generalizable predictor of prognosis and immunotherapy response. To facilitate the clinical adoption of the 3 S-MMR score, we developed an easy-to-use web tool for risk scoring as well as therapy stratification in LUAD patients. In summary, we have proposed and validated an ensemble learning model pipeline within the framework of metabolic reprogramming, offering potential insights for LUAD treatment and an effective approach for developing prognostic models for other diseases.

**Supplementary Information:**

The online version contains supplementary material available at 10.1186/s12967-024-05138-2.

## Introduction

Prior studies have shown that cancer cells frequently undergo epigenetic modifications to address challenges within the microenvironment and enhance their adaptability [1, 2]. Cell metabolism reprogramming, a vital tumor epigenetic change responding to varying energy demands [3], permits cancer cells to dynamically regulate bioenergetics and energy production by multiple metabolic pathways, including glycolysis [4], fatty acid metabolism [5], as well as lactic acid metabolism [6]. Malignant behaviors, such as proliferation, invasion, as well as metastasis, have been demonstrated to be associated with the reprogramming of cell metabolism, revealing a growing understanding of the underlying biological processes [7]. Although confirmed studies indicated alterations in the metabolic characteristics of LUAD cells via various metabolic pathways, leading to the induction of malignant behaviors as well as chemotherapy resistance [8, 9], the relationship between these alterations and patient prognosis has not been thoroughly elucidated. Furthermore, prior studies utilized tissue-level sequencing data constrained by cellular heterogeneity. Now, leveraging single-cell technology allows exploration of tumor characteristics at the single-cell level, effectively dissecting cellular heterogeneity and its impact on the tumor microenvironment (TME) [10]. However, the limited patient data from scRNA-seq samples has resulted in inadequate comprehensive studies on the relationship between metabolic reprogramming in cellular hierarchy structures and patient-to-patient variability, factors potentially responsible for the unfavorable prognosis and chemotherapy resistance in LUAD.

Historically, computational methods for predicting survival in LUAD patients have often relied on either single tumor-related pathways or individual machine learning algorithms or models, thereby severely constraining the robustness and precision of prognostication. Utilizing ensemble learning technique is an effective strategy to establish a robust model, as these methods have demonstrated superior accuracy compared to individual machine learning approaches across numerous biological prediction tasks, particularly in forecasting survival states. For instance, Kaur et al. devised an ensemble learning framework that incorporated support vector machine, random forest, as well as decision tree models as basic learners to predict the survival of ovarian cancer patients, achieved better predictive performance [11]. Zhu et al. collected glioma prognosis-related key genes and further constructed a high-performance ensemble learning model using GA [12]. Nonetheless, the precision as well as robustness of the ensemble learning model frequently encounter hurdles stemming from the feature engineering process and the variety in the basic learners. When basic learners and the ensemble learning model are concurrently trained on an identical cohort, there is a significant risk of overfitting, and the unique distributive traits of transcriptomic data, attributed to techniques like microarrays or RNA sequencing, further complicate the scenario [13]. Moreover, models constructed solely from bulk RNA-seq data tend to overlook the underlying biological rationale of the disease, lack adequate universality, and frequently encounter challenges in practical clinical applications.

In this study, we defined a set of genes correlated LUAD tumorigenesis and cell metabolism reprogramming named “MMR” according to LUAD scRNA-seq profiles. Machine leaning methods including Cox regression, random survival forest (RSF), CoxBoost, support vector machine (SVM), as well as Gradient Boosting Machine (GBM) were used to clarify the relationships between MMR and LUAD prognosis. We introduced an innovative ensemble learning pipeline, the three-stage MMR (3 S-MMR), augmented by genetic algorithm. This framework employs double training sets for feature engineering as well as model development, respectively, thereby mitigating the risk of severe overfitting. To bolster the robustness of the 3 S-MMR score, a novel gene-pairing method was implemented to remove the batch effects. Moreover, the integration of a GA automates the selection of basic learners within the ensemble learning model, facilitating the attainment of heightened accuracy [14]. The 3 S-MMR framework, which takes into account the fundamental biological aspects of cancer and metabolic reprogramming, has been validated as an important risk indicator in pan-cancer analyses. Subsequently, an easy-to-use web-tool has been developed to assist in predicting the future survival and guiding the therapy stratification of LUAD patients (https://xintisunlab.shinyapps.io/appLUAD/). Consequently, our study may offer insights for a deeper understanding of the genetic characteristics of metabolism in LUAD, as well as how metabolic reprogramming influences tumor malignancy and prognosis in LUAD patients.

## Methods and materials

### Data source

The scRNA-seq including 48 samples from normal lung (nLung, *n* = 11), normal lymph nodes (nLN, *n* = 10), early stage tumor lung (tLung, *n* = 11), advanced stage tumor lung (tL/B, *n* = 4), pleural fluids (PE, *n* = 5), as well as metastasis lymph node (mLN, *n* = 7) were downloaded from GEO database with accession ID: GSE131907 [15]. In total nine LUAD patient cohorts which contained overall survival (OS) information and expression data were collected. Among these cohorts, TCGA-LUAD obtained from the TCGA database, while GSE72094, GSE68465, GSE50081, GSE37745, GSE31210, GSE30219, GSE14814, and GSE3141 were downloaded from the GEO database. Immunotherapy cohorts: GSE126044 (NSCLC), GSE35640 (melanoma), GSE78220 (melanoma) cohorts and two scRNA-seq datasets: GSE207422 (NSCLC), and GSE145281 (Bladder Carcinoma) were also downloaded from the GEO database. One spatial transcriptomics sequencing (stRNA-seq) sample of LUAD with the accession ID GSE189487 was included in this study. The detailed information of all enrolled datasets is summarized in **Supplementary Table 1**. Metabolic gene sets were obtained from the KEGG, REACTOME, C5.BP, Hallmark MSigDB v5.2, and previous study [16].

### Data processing

For the processing of scRNA-seq data, we preserved high-quality cells that had fewer than 20% mitochondrial genes and expressed more than 200 genes. We also focused on genes that were expressed at levels between 200 and 7000 and were active in at least three cells. A total of 178,739 eligible cells were kept for further exploration. After that, the integration workflow conducted by Seurat pipeline [17]. The remaining cells were further scaled and normalized using a linear regression model with the “Log-normalization” method and the top 3000 highly variable genes were detected by the “FindVariableFeatures” function. Subsequently, the dimensionality of the scRNA-seq data was diminished through Principal Component Analysis (PCA). To remove the batch effects among the samples, soft k-means clustering was executed using the “Harmony” package [18]. The cell clustering was conducted using the “FindClusters” function, with the resolution parameter set at 0.8. The methodology for annotating cell clusters involved focusing on genes with elevated expression levels, genes exhibiting unique expression patterns, and documented canonical cellular markers. For Bulk-seq data, samples devoid of OS details were excluded. The TCGA-LUAD and GSE72094 cohorts were employed as training set 1 and training set 2, respectively, while all cohorts were amalgamated and defined as a meta set (*n* = 2066). We used gene-pairing method followed Eq. (1) to remove the batch effect.

1 $$Gene \left(ab\right)=\left\{\begin{array}{c}1, Ea>Eb\\ 0, Ea<Eb\end{array}\right.$$

### scRNA-seq analysis

To pinpoint malignant cells exhibiting clonal extensive chromosomal copy number variation (CNV), the CNV profiles of cells were deduced employing the package “inferCNV” [19]. The CNV score was formulated as the average of the squares of the ultimate CNV values for each chromosome. Labels indicating malignancy or non-malignancy were assigned by assessing the distribution of malignancy scores relative to the reference and identifying their bimodal characteristics. “FindMarkers” function were used to identify the differential expressed genes (DEGs) between malignant and normal cells. MMR gene set was quantified using AUCell, UCell, AddModuleScore, Singsore, and ssGSEA algorithms [20, 21].

### The pipeline of the 3 S-MMR ensemble learning model construction

In the creation of the 3 S-MMR score, a three-stage stepwise approach was employed. In the **Stage 1**, we performed feature engineering and gene-pairing to remove the batch effect in training set 1. In the **Stage 2**, to avoid over-fitting, training set 2 was applied to perform a 10-fold cross-validation (CV) as well as grid search before 47 basic learners were constructed. In the **Stage 3**, we applied training set 1 to establish the ensemble learning model, and GA was used to identify the optimal basic learner combination in the ensemble learning model.

### The details of pipeline are as follows

#### Stage 1: feature identification and engineering

A total of 1290 MMR genes were matched with expression data from 10 different cohorts using gene symbols, leading to a selection of 1066 genes for subsequent feature engineering. In the training set 1 (TCGA-LUAD cohort) for feature engineering, we first reduced the number of features using univariate Cox regression with a cutoff value of *P* < 0.05. Subsequently, the genes were paired as per Eq. (1) to remove the batch effect. To ensure adequate sample variation in the gene-pair features, only those gene pairs with a frequency ranging from 20 to 80% were retained in the training set 1. Afterward, we applied univariate Cox regression once again to select gene pair features (*P* < 0.05). Following this, we employed LASSO regression [22] for further screening, aiming to reduce the impact of multicollinearity on the results and identify gene pairs capable of predicting patients’ survival status.

### Stage 2: Basic learner construction

In the training set 2 (GSE72094 cohort), we conducted a 10-fold CV as well as grid search using five machine learning methods. For the 10-fold CV, training set 2 is partitioned into 10 folds, with the C-index and predicted values of the sub-model derived from each fold averaged to obtain the final values. Meanwhile, the predicted values for training set 1 are generated by the basic learners, and the resulting predictions are ultimately incorporated into the construction of the ensemble learning model in **Stage 3**. More precisely, in Cox models [23], the grid is selection = “forward”, “backward” or “both”; in RSF models [24], the nodesize is depth = 5, 10, 15 and 30, ntree = 50, 100, 200 and 500; in CoxBoost [25], the stepnos is depth = 50, 100, 150, 200, 250 and 300; in GBM models [26], the grid is depth = 1, 2, 5 and 10, ntree = 20, 50, 100 and 200; in SVM models, the grid is kernel = “linear”, gamma.mus = 0.1, 0.5, 1, 5, 10 and 50. Finally, we obtained 47 basic learners and they were sorted according to C-index [27] from high to low labeled as m*1*-m*47*.

## The details of machine learning methods of basic learners

In the Cox model, patient risk is influenced by two main factors: time and the patient’s own genetic characteristics. This model emphasizes the comparison of risks associated with these genetic characteristics. Moreover, we solely require the parameter *β* that maximizes the likelihood value of the current cohort event, as illustrated in Eq. (2):

2 $$1(\beta )=\sum\limits_{{i=1}}^{N} {\left[ {\beta \bullet X_{{}}^{{\left( i \right)}} - \ln \left( {\sum\limits_{{j:{t_j} \geqslant {t_i}}}^{{}} {\exp (\beta \bullet X_{{}}^{{\left( j \right)}})} } \right)} \right]}$$

In this equation, *N* and *X* represent the total number of samples and the number of features in each sample, respectively. The symbol *β* signifies the coefficient of the regression model, while *t* denotes the survival time. The Cox regression models were built using the “survival” and “survminer” packages.

The RSF model is a robust approach for predicting patient survival outcomes, leveraging an ensemble of Decision Trees (DTs). The model begins by employing the bootstrap method [28] to randomly sample subsets of the input data, typically using about two-thirds for training each tree, with the remaining one-third serving as out-of-bag (OOB) data for model validation. During the construction of each survival tree, features are selected randomly, enhancing the diversity and robustness of the model. A key aspect of RSF is the application of the Nelson-Aalen estimator for calculating the cumulative hazard function. This method is utilized to estimate the overall cumulative risk within the RSF model. The survival time (*T_i*) and status (*S_i*) of individuals, where 0 denotes censoring and 1 an event. The analysis at each terminal node of a decision tree involves assessing the number of events (deaths) *D*_*(i, h)*_ and the total number of individuals *Y*_*(i, h)*_ present. Utilizing the Nelson-Aalen estimator [29], the cumulative risk function of a single terminal node can be obtained, as shown in Eq. (3).

3 $${H^ \wedge }_{h}\left( T \right)=\sum\limits_{{{T_{ih}} \leqslant T}}^{{}} {\frac{{{D_{\left( {i,h} \right)}}}}{{{Y_{\left( {i,h} \right)}}}}}$$

Define “*C*_(*i, b*)_” as a binary indicator for each sample, where “*C*_(*i, b*)_ = 1” if the ith sample is part of the in-bag data for the bth bootstrap sample or tree, and “*C*_(*i, b*)_ = 0” if it is an out-of-bag sample. “$H_{b}^{*}\left( {t| {X_i}} \right)$” represents the ith out-of-bag sample. The total cumulative risk for the model is then calculated as outlined in Eq. (4).

4 $${\rm H}_{E}^{{^{{ * * }}}}=\frac{{\sum\nolimits_{{b=1}}^{B} {{C_{i,b}}} H_{b}^{*}\left( {t| {X_i}} \right)}}{{\sum\nolimits_{{b=1}}^{B} {{C_{i,b}}} }}$$

The accuracy of the model can be evaluated using the C-index, as depicted in Eq. (5).

5 $${\text{C-index}}= \frac{{N\left( {{\text{sample pairs predicted correctly}}} \right)}}{{N\left( {{\text{sample}}} \right)}}$$

In the context of the SVM model [14], the primary objective is to find the optimal hyperplane that maximizes the margin between the nearest samples of different classes, known as support vectors, and the hyperplane itself. This margin is crucial as it determines the classifier’s generalization ability. The distance *d*, which refers to the margin between the support vectors and the classification hyperplane, is quantified by Eq. (6).

6 $${\text{d}}\left( {w,b} \right)={\hbox{min} _{xi,yi= - 1}}d\left( {w,b;{x_i}} \right)+{\hbox{min} _{xi,yi=1}}d\left( {w,b;{x_i}} \right)=\frac{2}{{||w||}}$$

Here, *x* denotes the input feature vectors, *w* is the vector of model weights, and *b* signifies the bias term. The term || *w* || is the L2 norm of the weight vector, which is central to calculating the margin. The expression above is equivalent to the minimized objective function presented in Eq. (7). The constraints are defined in Eq. (8).

7 $$\hbox{min} \frac{1}{2}{w^T}w$$

8 $${y_i}\left( {{w^T}{x_i}+{\text{b}}} \right) \geqslant 1$$

To allow the SVM model to address linearly inseparable tasks, a slack variable (**ξ**) and a penalty factor (*C*) are introduced, as depicted in Eq. (9). If the slack variable is non-zero, it indicates that the adjusted sample violates the inequality constraint. The penalty factor (*C*) is a manually set parameter greater than 0, employed to penalize samples that breach the inequality constraints. Equation (10) presents the modified constraint condition for the soft-margin SVM, accounting for the slack variables (**ξ**_***i***_). This constraint ensures that each data point *x*_*i*_ with label *y*_*i*_ will be on the correct side of the margin, allowing for some misclassifications as controlled by the slack variables. Additionally, the SVM models are constructed using the “survivalsvm” package.

9 $$\hbox{min} \frac{1}{2}{w^T}w+C\sum\limits_{{i=1}}^{N} {{\upxi _i}}$$

10 $${y_i}\left( {{w^T}{x_i}+b} \right) \geqslant 1 - {\upxi _i}$$

The GBM model [30] employs DTs as its fundamental learners. It is an ensemble method where the final model is constructed in a stepwise manner through the additive combination of individual trees and be expressed as Eq. (11): individual trees denoted as T(*X*, *θ*_*m*_), and *θ*_*m*_ represents the set of parameters for the m-th tree out of a total of *M* trees. Equation (12) detailing the update mechanism at each step.

11 $${f_M} ( X )=\sum\limits_{{m=1}}^{M} {T( {X,{\uptheta _m}} )}$$

12 $${f_M}\left( X \right)={f_{m - 1}}\left( X \right)+T\left( {X,{\uptheta _m}} \right)$$

where the *f*_m−1_(*X*) is the previous model, and the parameters of the current model are determined through empirical risk minimization of *θ*_*m*_, as shown in Eq. (13).

13 $$\uptheta _{m}^{ \wedge }=\arg \hbox{min} \sum\limits_{{i=1}}^{N} {L\left( {{y_i},{f_{m - 1}}\left( X \right)+T\left( {X,{\uptheta _m}} \right)} \right)}$$

In this context, “*L*” denotes the loss function, which quantifies the difference between the predictions and the true values. The expressions “*f*_m−1_(*X*)” represent the model’s predictions from the previous iteration, and “*y*_*i*_” are the observed target values. The current model, *T* (*X*, *θ*_*m*_​), is designed to adjust the predictions by fitting to the residuals—differences between observed values and the previous model’s predictions—to minimize the overall loss within the GBM framework.

The CoxBoost model modifies the Cox proportional hazards model by incorporating a penalty term to the partial likelihood. The iterative process can be conceptually represented by the Eq. (14).

14 $$h(t|x)={h_0}(t){\text{exp}}(\sum\nolimits_{{j=1}}^{p} {{\beta _j}{x_j}+{\text{Penalty(}}\beta ,\lambda {\text{)}}} )$$

where *h*(*t*∣*x*) is the hazard function at time *t* given covariates *x*, *h*_0​_(*t*) is the baseline hazard function, *β*_*j*_​ are the coefficients, *x*_*j*​_ are the covariates, and Penalty (*β*, *λ*) represents the penalization term that is applied to the coefficients to prevent overfitting. The penalization term is typically a function of the coefficients *β* and a regularization parameter *λ*, which is adjusted throughout the boosting process.

### Stage 3: ensemble learning model construction via genetic algorithms

The 47 basic learners (m*1*-m*47*) were applied to develop the ensemble learning models in training set 1. The ensemble learning model’s methods and parameters align with those of *m1* (RSF, nodesize = 30, ntree = 100). Specifically, we adopted a “stacking” approach to construct the ensemble learning model, where the input data comprises the predicted risk scores generated by these 47 basic learners. It’s crucial to note that, in order to guarantee the predictive performance of the ensemble learning model, the selection of basic learners must consider both model accuracy as well as model diversity. GA [14, 31] mimics the natural process of chromosomal recombination evolution and has been demonstrated to be well-suited for optimization problems related to genomics. To streamline and automate this process, we utilize the “GA” package to optimize the selected basic learners. It should be noted that GA consists of the fitness function, selection operator, crossover probability, and mutation probability.

In our pipeline, ensemble learning models with diverse basic learners’ combinations can be treated as separate entities within the GA. In fact, each specific individual corresponds to a one-hot vector, where “1” signifies the inclusion of the respective sub-model in the ensemble, and “0” signifies its exclusion. Our objective is to identify the best basic learners’ combination by assessing these individuals. Furthermore, a fitness function was applied to evaluate individuals, as depicted in Eq. (15), noting that a lower fitness value indicates a better combination of basic learners.

15 $$f\left( {{x_i}} \right)= \left\{ {_{{1 - {\text{Cindex}}\left( {{X_i}} \right),{N_{{\text{sub-model}}}}{\text{in individual }}{{\text{x}}_i} \geqslant 2}}^{{{\text{ 1}},{N_{{\text{sub-model}}}}{\text{in individual }}{{\text{x}}_i}<2}}} \right\}$$

Initially, we chose pairs of individuals that require crossing over in the new generation during the evaluation process using the selection operator. In pursuit of this goal, we examined three different selection operators, comprising *Roulette* (Eq. (16)), *Liner rank* (Eq. (17)) as well as *Tournament*. Specifically, within the *Roulette* operator mechanism, the probability of selecting an individual is determined by comparing its fitness value with the aggregate fitness of the whole population (Eq. (16)). In the mentioned selection methods, default settings from the “genalg” and “GA” packages were employed. Regarding the *Tournament* operator, q individuals were randomly chosen from the population for comparison. Subsequently, the individual with the best fitness value was considered the parent of the next generation. We set *q* = 2, which is the default parameter in the “GA” package.

16 $${{\text{P}}_{{\text{selection}}}}=\frac{{f\left( {{x_i}} \right)}}{{\sum\nolimits_{{{\text{n}}={\text{1}}}}^{{{{\text{N}}_{i{\text{ndividual}}}}}} {f\left( {{x_n}} \right)} }}$$

17 $${{\text{P}}_{{\text{selection}}}}={{\text{P}}_{\hbox{min} }}+\left( {{{\text{P}}_{\hbox{max} }} - {{\text{P}}_{\hbox{min} }}} \right)\frac{{i - 1}}{{N - 1}}$$

Subsequently, the selection of crossover locations involves generating a random integer I (where 1 < I < 47), aligning with a crossover likelihood of 0.8 with default parameter. After the evaluation of a generation is completed, each individual has a mutation probability denoted as *M*, which is calculated as shown in Eq. (18):

18 $$M=\frac{1}{{{N_{{\text{individual}}}}}}$$

When an individual undergoes mutation, *N*_*mg*_ randomly chosen digits in it will alter. Concurrently, selection pressure was assessed through takeover time, indicating how long it takes for the fittest individual to dominate the population, with longer times implying lower selection pressure. Ultimately, the individual with the smallest fitness value was deemed the fittest. From this individual’s chosen basic learner combination, we constructed the ensemble model 3 S-MMR score using training set 1.

### Assessment of the 3 S-MMR score

To rigorously assess the robustness as well as predictive accuracy of the 3 S-MMR score, we undertook a multifaceted analytical approach encompassing Kaplan-Meier (KM) analysis, ROC-AUC, as well as C-index calculations. In the KM analysis, patients were categorized into high 3 S-MMR score group and low 3 S-MMR score group according to the median values identified across 10 distinct cohorts. Statistical substantiation of the differentiation was done using the Log-Rank test. The ROC-AUC analysis was conducted to evaluate the performance of the model, where the AUC value was calculated to assess the diagnostic ability of the model. Further comparisons were made between the 3 S-MMR score and ensemble learning baseline models without GA stage, top 5 and top 10 model in CV, subsequently, as well as with the C-index of commonly used clinical indicators. This comprehensive analysis delineated the efficacy of the 3 S-MMR score in prognostic predictions, showcasing its reliability and accuracy.

### Development of nomogram and web-tool

To facilitate the use of the 3 S-MMR score among clinicians as well as researchers, we devised a nomogram and a web-tool, integrating several clinical variables to surpass the accuracy of the 3 S-MMR score. Initially, clinical factors along with the 3 S-MMR score underwent univariate Cox regression analysis to identify elements predictive of survival outcomes. Subsequently, a multivariate Cox regression was performed, ensuring the selection of factors devoid of significant collinearity. Web-tool was developed using “shiny” and “shiny dashboard” packages [32].

### Cell-cell interaction, SCENIC, and trajectory analysis

Utilizing the “cellchat” package [33], the communication patterns among cells were discerned by inferring, scrutinizing, and graphically representing the receptor-ligand signaling interactions between malignant cells of both high and low 3 S-MMR score groups, and elucidating the contributions of diverse 3 S-MMR score groups within particular pathways. The analysis of gene regulatory networks was carried out utilizing the “SCENIC” package [34] to identify networks based on the co-expression of gene regulons and DNA motifs. Specifically, the co-expression network was calculated by *runGenie3* as well as the regulons were identified by *RcisTarget*. *AUCell* was performed to calculate the TF regulons’ activity. We also used Monocle2 [35] to infer the cell trajectory. Following dimension reduction and cell sequencing, the trajectory of differentiation was deduced using the standard parameters. We also performed CytoTRACE analysis with default parameter [36, 37], an algorithm that predict differentiation states from scRNA-seq data based on the simple yet robust observation that transcriptional diversity decreases during differentiation, to complement the trajectory analysis form Monocle2.

### Virtual simulation for the prediction of therapeutic targets and agents in silico

The expression profile data of human cancer cell lines (CCLs) were downloaded from the CCLE project [38] (https://portals.broadinstitute.org/ccle/). CERES scores, derived from genome-wide CRISPR knockout screenings encompassing 18,333 genes across 739 cell lines, were obtained from the DepMap portal (https://depmap.org/portal/). It is pertinent to acknowledge that the CERES score assesses the dependence level of a targeted gene in a specific Cancer Cell Line (CCL), with a lower score signifying a greater probability of the gene’s essentiality in the proliferation and sustenance of that CCL. Considering that many proteins do not possess binding sites or sufficient affinity for small molecules or antibodies, we initially identified 2249 druggable targets from a previous publication [39]. For identifying potential therapeutic agents, we initially harnessed the Connectivity Map (CMap, https://clue.io/query). This tool juxtaposes a differential gene signature against all perturbation signatures in CMap, yielding a score that evaluates the congruence between these signatures [40, 41]. Hence, medications with scores below − 95 are deemed suitable candidates for counteracting the differential signatures of the 3 S-MMR score. Utilizing the publicly accessible Genomics of Drug Sensitivity in Cancer (GDSC, https://www.cancerrxgene.org/) database, the chemotherapy response for each sample was predicted. The prediction process was conducted using the “oncoPredict” package [42], which involves the calculation of drug sensitivity values akin to the half-maximal inhibitory concentration (IC50). The candidate agents for LUAD patients with high 3 S-MMR score was also generated from the Cancer Therapeutics Response Portal (CTRP; https://portals.broadinstitute.org/ctrp) as well as Profiling Relative Inhibition Simultaneously in Mixtures (PRISM; https://depmap.org/portal/prism) databases.

### Pan-cancer analysis

To delve deeper into the potential of the 3 S-MMR score to forecast survival outcomes across various cancer types, we retrieved expression data as well as OS information from the TCGA database for a cohort of 10,110 patients spanning 33 different types of cancer. The detailed of 33 cancer cohorts are provided in the **Supplementary Table 1.**

### Statistical analysis

Data processing, statistical analyses, and graph plotting were all performed using R software, version 4.2.1. Relationships between continuous variables were assessed with Spearman’s correlation coefficients, while chi-squared tests were employed for categorical data comparisons. Depending on their distribution, continuous data were analyzed using either Wilcoxon rank-sum tests or student’s T tests. Parameters for R packages were described in the corresponding sections, with default settings applied unless otherwise specified. All tests were conducted bidirectionally with a significance threshold set at *P* < 0.05.

## Results

### Cellular dynamics changing across early, advanced, and metastatic LUAD

**Fig. 1:**
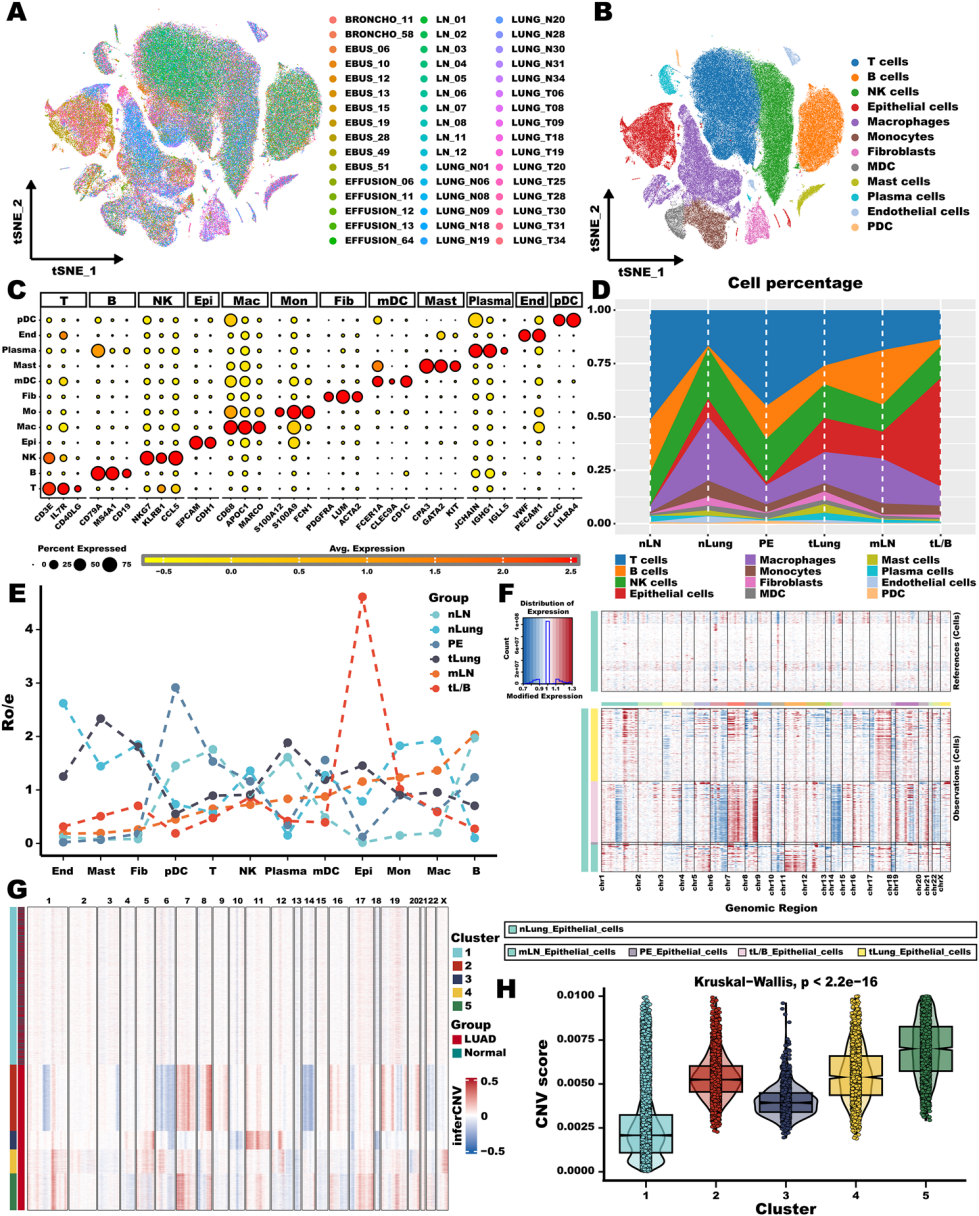
**Cellular dynamics changing across early, advanced, and metastatic LUAD.** (**A**) The cell distribution of the samples showed no significant batch effect. (**B**) The t-SNE map of cells from all scRNA-seq samples, colored by cell-type annotation. (**C**) Dot plot showing representative marker genes for each cell type. (**D**) The proportion of each cell type from tissues of each origin, as indicated. (**E**) Line chart showing tissue prevalence for each cell type estimate by Ro/e score. (**F**) Graded heatmap showing CNVs of epithelial cells from tissues of each origin. Normal lung origin epithelial cells are used as a control reference. Red: gains; blue: losses. (**G**) K-means clustering of inferred CNVs to obtain cancer cells. (**H**) Violin plot showing the difference in CNV score of the five K-means clusters

After applying the harmony algorithm, the cellular distribution within each sample exhibited overall consistency, suggesting the absence of significant batch effects among the samples, which could be utilized for downstream analysis (Fig. 1A). In the initial stage of unraveling the organization of cellular hierarchies in LUAD, we re-analyzed the 178,739 scRNA-seq cells covering 48 samples including Nln, nLung, tLung, PE, mLN, and tL/B tissues, as well as notably classified T, B, NK, epithelial, macrophages, monocytes, fibroblasts, MDC, mast, plasma, endothelial, and PDC according classical marker genes (Fig. 1B). The marker genes pertaining to each cell population demonstrated conspicuous distinctions, signifying annotation accuracy (Fig. 1C). The most pronounced shifts in in terms of the population of cell types were observed in epithelial, T, and macrophages among different types (Fig. 1D). We further quantified the tissue enrichment of all cell populations according to Ro/e analysis [43]. Within the entirety of cell populations, epithelial cells exhibited a notable preference for distribution in the tL/B, with the subsequent highest presence observed in tLung and mLN tissue origins (Fig. 1E). LUAD is largely driven by changes in gene copy number; consequently, we then analyzed scRNA-seq data to infer copy number variations in cancer cell populations. The result of CNV profiles showed inter- and intra-lesion heterogeneity in primary and metastasis tissues (Fig. 1F). Unsupervised clustering based on K-means using 5 clusters was applied to distinguished between cells with high and low CNV (Fig. 1G). The CNV scores of clusters 1 was at the bottom, as it contained more normal tissue original epithelial cells (Fig. 1H). Hence, cluster 1 was designated as normal epithelial cells, while the remaining cells were classified as malignant cells.

### Heterogeneity among LUAD driven malignant metastasis mediated by cell metabolism reprogramming

**Fig. 2:**
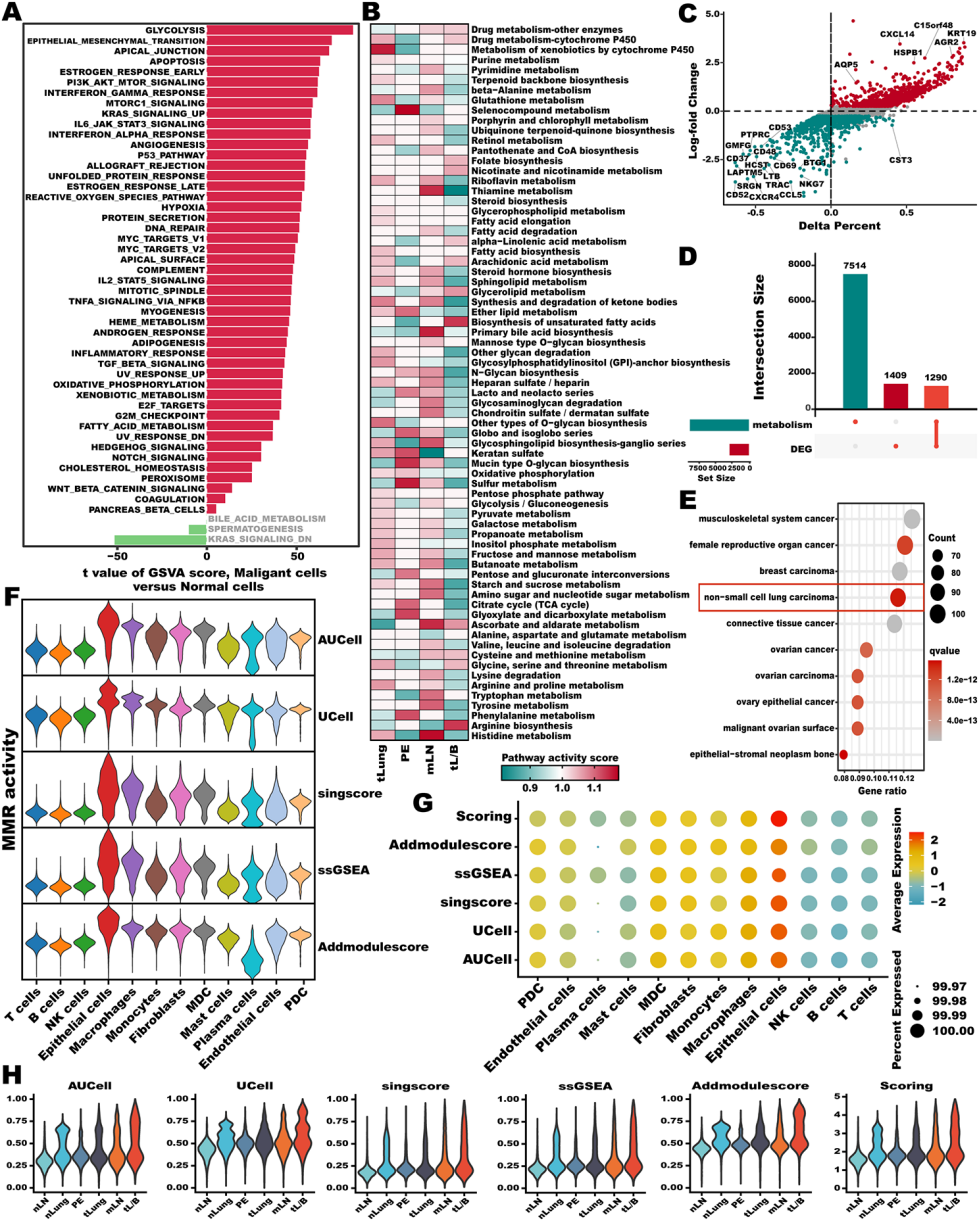
**Heterogeneity among LUAD driven malignant metastasis mediated by cell metabolism reprogramming.** (**A**) Differences in hallmark gene set pathway activities scored by per cell by GSVA between normal and malignant cells. (**B**) Metabolic pathway activities in malignant cells from tissue of each origin. Statistically non-significant values (random permutation rest *P* > 0.05) are shown as blank. (**C**) Percentage difference (Delta means percent of cells) and log-fold change based on the Wilcoxon rank-sum test results for differential expressed genes between malignant and normal cells. (**D**) UpSet plot showing the intersection analysis of the 1290 MMR genes. (**E**) DO enrichment analysis of 1290 MMR genes. (**F, G**) Violin plot (**F**), and Bubble plot (**G**) showing enrichment scores of MMR gene sets for each cell type using AUCell, UCell, singscore, ssGSEA, AddModulescore, and Scoring (the sum of scores from other algorithms) score. (**H**) Violin plot showing the dynamic change of enrichment scores of MMR gene sets for tissues of each origin using AUCell, UCell, singscore, ssGSEA, AddModulescore, and Scoring score

Analysis of hallmark pathway underscored that more changes were between non-malignant cells and malignant cells (Fig. 2A). A direct comparison of malignant versus normal cells revealed glycolysis as the top enriched signature in malignant cells, many metabolism-related pathways such as heme metabolism, xenobiotic metabolism, as well as fatty acid metabolism also activated in malignant cells (Fig. 2A). Afterwards, the scMetabolism pipeline [44], focusing on metabolic changes, indicated a completely different activation of metabolic pathways between malignant and normal cells, which suggested that cell metabolism reprogramming was a significant explanation for LUAD metastasis (**Supplementary Fig. 1**). We next sought to identify the overall features of metabolic pathway variation among the malignant cells form primary and metastatic samples from LUAD patients. Following the protocol outlined in the study by Xiao et al. [45], we defined the pathway activity score as the average of the relative gene expression values of all genes within a single metabolic pathway and across all cells of a specific type. We found discernible variations in metabolic activities across malignant cells at varying tumor stages, underscoring the pivotal role of metabolic reprogramming in tumor evolution and substantiating its critical significance in the intricate process of tumor progression (Fig. 2B). We further identified the DEGs between the malignant and normal cells (Fig. 2C, **Supplementary Table 2**). Consequently, we intersected metabolism-related pathways genes form KEGG, GO, REACTOM, published literatures and above DEGs generated a set of genes named Malignant & Metabolism Reprogramming (MMR) (Fig. 2D, **Supplementary Table 3**). Disease ontology enrichment analysis confirmed that MMR genes were significantly enriched in cancer, especially in non-small cell lung cancer (Fig. 2E). To investigated whether MMR activity was dynamic in metastasis population, we further quantified MMR scores in scRNA-seq level using AUCell, UCell, AddModuleScore, singsore, and ssGSEA algorithms. All algorithms indicated that MMR score was higher in the epithelial cells, macrophages, fibroblasts, while lower in the T, B, and NK cells (Fig. 2F and G). With the tumor progression and metastasis, the MMR score exhibited a significant dynamic increase, indicating that MMR is involved in the progression and metastasis of LUAD (Fig. 2H).

### 3 S-MMR score pipeline construction

#### Stage 1: prognosis-related MMR feature engineering

**Fig. 3:**
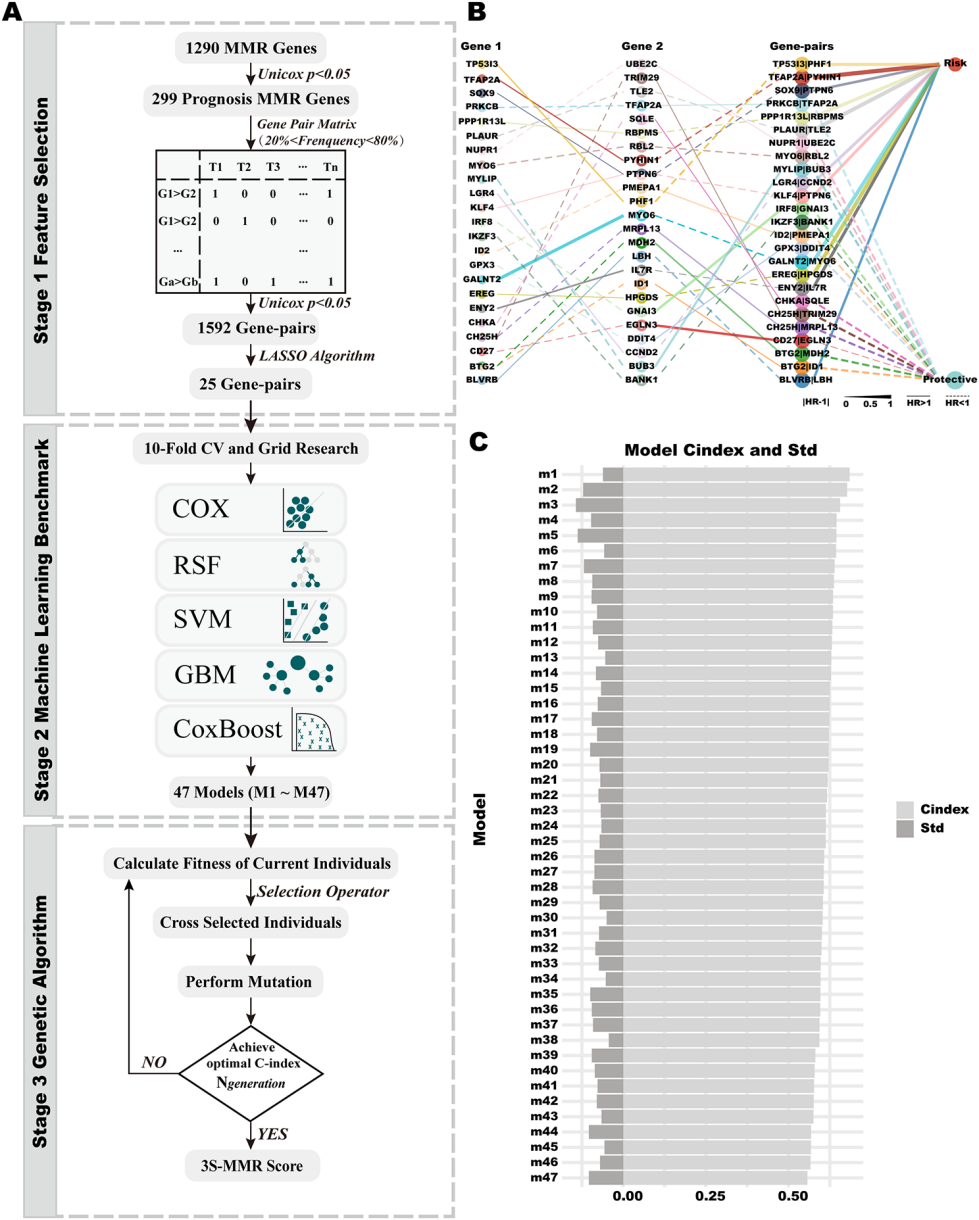
**3 S-MMR score pipeline construction.** (**A**) Workflow of the 3 S-MMR score. (B) The gene-pair information and hazard ration of 25 LASSO driverd gene-pairs. (C) The C-index and Std of 47 basic learners

The overall workflow of the 3 S-MMR score is displayed in Fig. 3A. For the establishment and validation of the 3 S-MMR score, we gathered data from 9 cohorts of LUAD patients, comprising a total of 2066 individuals with comprehensive genome-wide transcriptomic profiles and OS data. Specifically, the TCGA cohort (*n* = 500) and GSE72094 cohort (*n* = 398) were applied as **training set 1** and **training set 2**, respectively. In order to achieve precision in predicting the prognosis of LUAD patients, our initial step involved the application of univariate Cox regression (*P* < 0.05) to screen and retain 299 MMR gene sets as essential features (**Supplementary Table 4**), as well as paired these genes to obtained 3080 gene-pairs whose frequency was between 20 − 80% (Eq. 1). Furthermore, univariate Cox regression (*P <* 0.05) was performed to reduce theses gene pairs to 1592 gene-pairs (**Supplementary Table 5**). Following that, LASSO was employed, and when the partial likelihood of deviance reached its minimum, 25 gene pairs were retained (**Supplementary Table 6**, Fig. 3B), as fundamental features for constructing basic learners in **Stage 2**.

### Stage 2: Basic learner construction

To prevent severe overfitting, we used the GSE72094 cohort as training set 2 instead of the TCGA cohort, conducting 10-fold CV, grid search, and establishing basic learners in **Stage 2**. We applied five machine learning algorithms for the creation of basic learners, and comprehensive descriptions of these methods can be found in the “**methods**” section. During this stage, 47 basic learners were yielded, and the RSF demonstrated a superior predictive effect (Fig. 3C, **Supplementary Table 7**). Ultimately, the scores predicted by these basic learners were utilized as input data for training the ensemble learning model in **Stage 3**.

#### Stage 3: ensemble learning model construction

Given the importance of both diversity and accuracy of basic learners for the performance of the ensemble learning model, we assessed the accuracy of basic learners using the C-index in CV. GA is an optimization approach rooted in natural genetic mechanisms, employs a randomized yet directed search mechanism for discovering the global optimal solution. To automate and enhance this process, we employed GA to optimize the combination of basic learners in the **training set 1** (TCGA cohort). In this task, the global optimal solution involves discovering the best combination of basic learners, with the fitness value represented by Eq. (15) serving as the optimized function. Subsequently, it was determined that the optimized basic learner combination comprised 26 basic learners (**Supplementary Table 7**). Ultimately, the 3 S-MMR ensemble learning model was constructed.

### The evaluation of the 3 S-MMR score

**Fig. 4:**
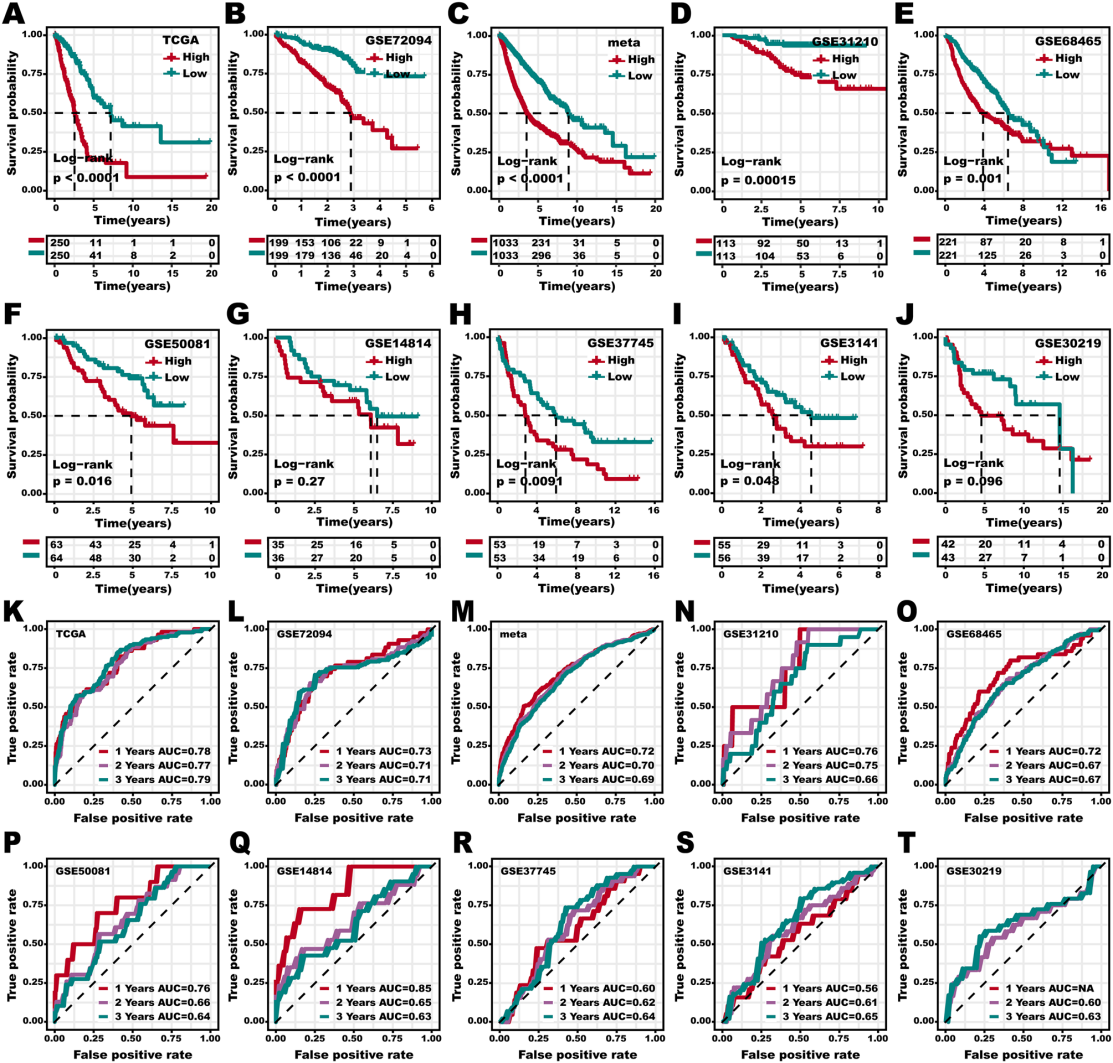
**Evaluation of 3 S-MMR score. (A-J)** Kaplan-Meier survival curves of 3 S-MMR score in the training set 1 (TCGA-LUAD) (**A**), training set 2 (GSE72094) (**B**), meta (**C**), GSE31210 (**D**), GSE68465 (**E**), GSE50081 (**F**), GSE14814 (**G**), GSE37745 (**H**), GSE3141 (**I**), GSE30219 (**J**) cohorts. **(K-T)** ROC curves of 3 S-MMR score in the training set 1 (TCGA-LUAD) (**K**), training set 2 (GSE72094) (**L**), meta (**M**), GSE31210 (**N**), GSE68465 (**O**), GSE50081 (**P**), GSE14814 (**Q**), GSE37745 (**R**), GSE3141 (**S**), GSE30219 (**T**) cohorts

To evaluate the prognostic performance of the 3 S-MMR score, we employed it to 10 LUAD patient cohorts. Noteworthy, the 3 S-MMR score demonstrated robust prognostic value in distinguishing the survival status of LUAD patients, not only in training set 1 (TCGA-LUAD, Fig. 4A), training set 2 (GSE72094, Fig. 4B), meta cohort (Fig. 4C), as well as seven LUAD independent test cohorts, respectively (Fig. 4D and J). ROC curve analysis revealed high AUC values for the 3 S-MMR score in predicting prognosis among 10 independent LUAD patient cohorts, respectively (Fig. 4K and T). These findings indicated that the 3 S-MMR score, which incorporates fundamental metabolic reprogramming, gene-pairing, double training sets, as well as GA, serves as a wide prognostic indicator across diverse LUAD cohorts. This remains true despite variations in transcriptomic platforms, clinical attributes, and genetic profiles within these LUAD cohorts, including for new patients.

#### Comparison of 3 S-MMR score to clinical variables and web-tool development

**Fig. 5:**
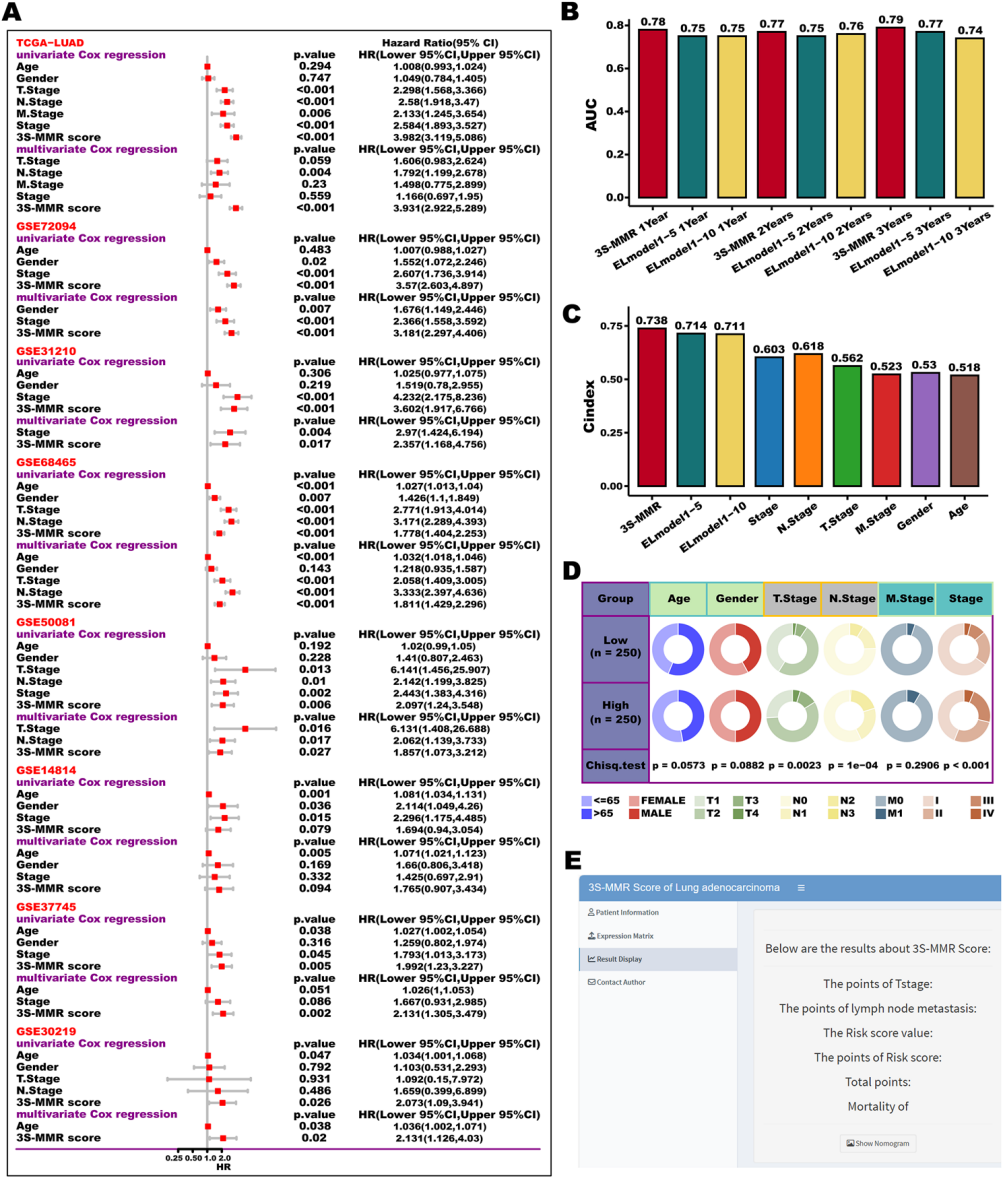
**The evaluation of the 3 S-MMR score and web-tool development.** (**A**) Forest plots demonstrate the hazard ratio (HR), 95% confidence interval (CI), and the corresponding *P* values of both univariate Cox regression analysis and multivariate Cox regression analysis in 8 LUAD cohorts. (**B**) Comparison of time-dependent area under the receiver operating characteristic curve (AUC) values at 1-, 2-, and 3-year among the 3 S-MMR score and ensemble baseline models. (**C**) Comparison of C-index between models and clinical features in the TCGA cohort. (**D**) Circos plot of different clinical features in high and low 3 S-MMR score groups. (**E**) The page of 3 S-MMR score web-tool

Clinical variables such as TNM staging as well as Grade are commonly applied to guide LUAD management as well as predict prognosis, we then conducted univariate and multivariate Cox regression analyses. The result suggested that 3 S-MMR score as a continues variable was significantly associated with a shorter OS time in all cohorts and was considered an independent risk factor for LUAD prognosis (Fig. 5A). In addition, we excluded the GA stage and considered the top 5/10 basic learners in CV average risk scores as two types of baseline ensemble learning models. We then compared two ensemble learning baseline models and proposed 3 S-MMR score using the ROC-AUC analysis (Fig. 5B). The high AUC values of 3 S-MMR score showed better predictive accuracy than ensemble baseline models in different time-point. We further compared clinical features, ensemble baseline models, and 3 S-MMR score using C-index (Fig. 5C**).** Overall, 3 S-MMR score displayed better predictive accuracy than clinical features and ensemble baseline models. Additionally, the 3 S-MMR score exhibited significant associations with survival status, tumor stage, and the TNM staging system in the TCGA-LUAD cohort (Fig. 5D). Recognizing the 3 S-MMR score’s superior predictive capability for LUAD patient survival, our aim was to enhance its usability for the research community. To realize this, we designed a pertinent nomogram and a web tool, which are presented in Fig. 5E and accessible through the following link: (https://xintisunlab.shinyapps.io/appLUAD/).

#### Ablation experiments

**Fig. 6:**
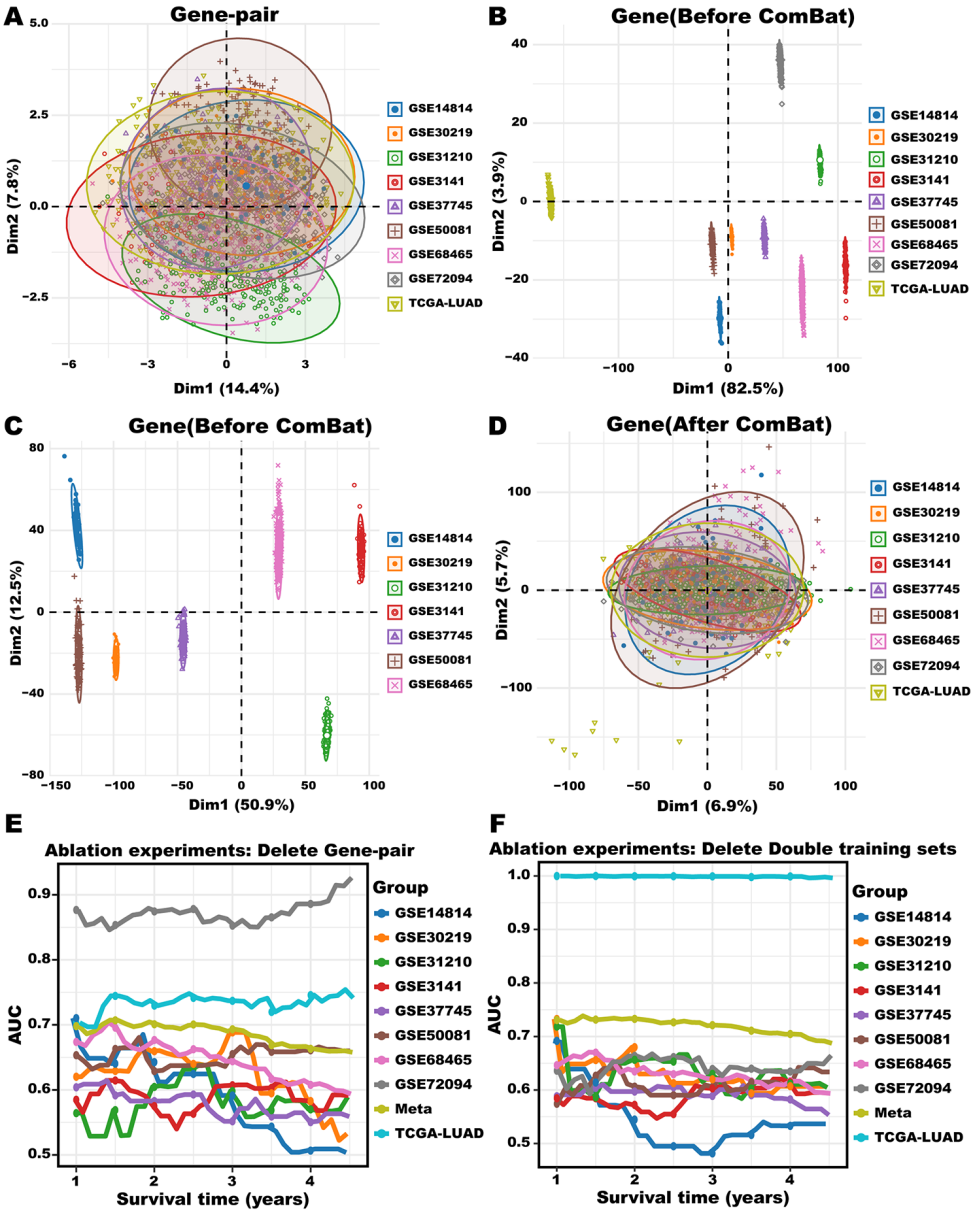
**The ablation experiments of the 3 S-MMR score.** (**A**) PCA analysis of gene-pair features in 9 LUAD cohorts. (**B**) The PCA analysis of unpaired gene features in 9 LUAD cohorts. (**C**) The PCA analysis of unpaired gene features in 7 LUAD cohorts. (**D**) The PCA analysis of unpaired gene features in 9 LUAD cohorts after using ComBat algorithm. (**E**) ROC curves of 3 S-MMR score in 10 cohorts without gene-pairing method. (**F**) ROC curves of 3 S-MMR score in 10 cohorts without double training sets

To illustrate the robustness of the gene-pairing method, we conducted a comparison with unpaired gene features using principal component analysis (PCA) (Fig. 6A and D). It’s worth mentioning that the distributions of PC1 and PC2 for gene-pair features were similar across nine cohorts (Fig. 6A). However, the PC1 and PC2 of TCGA-LUAD and GSEE72094 cohorts without gene-pairing significantly deviated from other cohorts (Fig. 6B). Even after removing these two cohorts and conducting PCA again with unpaired gene features, we still found obvious distributions in PC1 and PC2 among the remaining cohorts (Fig. 6C). Furthermore, we applied the widely-used Combat algorithm [46] to reduce the batch effect on raw data, we found that the batch effect would be reduced (Fig. 6D), which showed a similar condition with that in Fig. 6A, but the raw expression data were changed. In summary, our results demonstrate that the gene-pairing method effectively mitigates batch effects while preserving the distribution of raw data. Afterward, in order to demonstrate the necessity of using the gene-pairing method and double training sets for the 3 S-MMR pipeline, we further performed ablation experiments [47]. When using gene features directly instead of gene-pairing features, we employed the same feature selection methods. Building upon the foundation of 299 prognosis-related MMR genes, we applied LASSO regression to further refine the selection, ultimately obtaining 31 features (**Supplementary Table 8**). Next, we constructed the new 3 S-MMR score again according these 31 features. It is noted that the ROC-AUCs of the new 3 S-MMR score were lower than the original 3 S-MMR score with gene-pairing used (Fig. 6E, **Supplementary Fig. 2**). Besides, the Kaplan-Meier analysis indicated that only 5 cohorts showed a statistically significant difference in OS (**Supplementary Fig. 2**). Furthermore, during the process of verifying the importance of double training sets, we only used TCGA-LUAD cohorts as the training set to see whether double training sets can effectively avoid serve over-fitting, finally re-constructing the 3 S-MMR score. When applied to a single training set, it implies that the entire training process would be conducted exclusively within the TCGA-LUAD dataset, encompassing whole three stages. Interestingly, we observed that the ROC-AUC values of the training set were close to 1.0, while noting that the ROC-AUCs of other cohorts were obviously lower than that of the original 3 S-MMR score, indicating that the model is experiencing severe overfitting (Fig. 6F, **Supplementary Fig. 3**). Subsequently, the Kaplan-Meier analysis indicated only 4 cohorts obtained statistically significant difference in OS (**Supplementary Fig. 3**). This result proved that the application of double training sets contributed to avoiding severe over-fitting. Taken collectively, our results proved that it is necessary to perform the gene-pairing method, double training sets, and GA to make sure the robustness and high accuracy of the 3 S-MMR score.

#### 3 S-MMR score remolds the immune infiltration patterns in LUAD

**Fig. 7:**
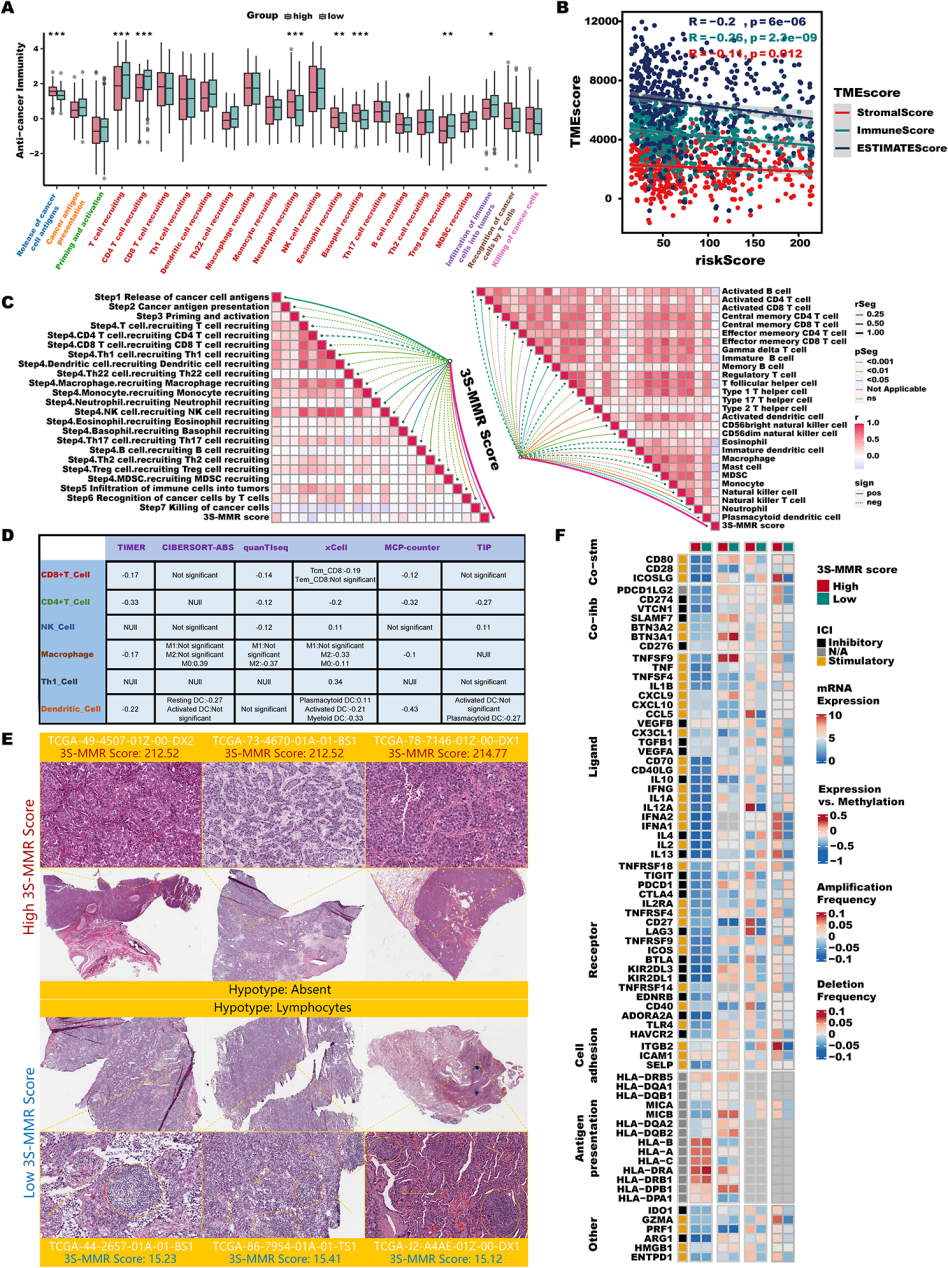
**3 S-MMR score remolds the immune infiltration patterns in LUAD.** (**A**) Differences in the various steps of the cancer immunity cycle between high- and low-3 S-MMR score groups. (**B**) Correlations between 3 S-MMR score (riskScore) and stromal, immune, and ESTIMATE score. (**C**) Correlations between 3 S-MMR score and the steps of the cancer immunity cycle (left). Correlations between 3 S-MMR score and the enrichment scores of published immune cell signatures (right). (**D**) Correlation between 3 S-MMR score and the infiltration levels of six types of TIICs (CD8 + T cells, CD4 + T cells, NK cells, macrophages, Th1 cells, and dendritic cells), which were calculated using six independent algorithms. (**E**) Image representing the pathological HE staining variation between the high- and low-3 S-MMR score groups (TCGA database). (**F**) From left to right: mRNA expression (median normalized expression levels); expression versus methylation (gene expression correlation with DNA-methylation beta-value); amplification frequency (the difference between the fraction of samples in which an IM is amplified in a particular subtype and the amplification fraction in all samples); and the deletion frequency (as amplifications) for 75 IM genes by the high- and low-3 S-MMR score groups. Abbreviation: *P < 0.05; **P < 0.01; *** *P* < 0.001

The immune landscape of the TME in LUAD is characterized by several factors: the presence of immunomodulatory molecules, the dynamic processes of the cancer immunity cycle, the degree of Tumor-Infiltrating Immune Cells (TIICs) penetration, and the levels of inhibitory immune checkpoint expression. Therefore, we further delved into these aspects to thoroughly analyze the relationship of 3 S-MMR score with immune regulation. The dynamics of the cancer immunity cycle represent a direct and holistic manifestation of the roles played by the chemokine system and various other immunomodulators (IMs) [48, 49]. In the low 3 S-MMR score group, activities of the majority of the steps in the cycle were identified as being significantly elevated, including the release of T cell recruiting (Step 4) and Infiltration of immune cells into tumors (Step 5), while release of cancer cell antigens (Step 1) was upregulated in high 3 S-MMR score group (Fig. 7A). Furthermore, ESTIMATE algorithm confirmed that 3 S-MMR score was significantly negatively correlated with stromal, immune, and ESTIMATE score (Fig. 7B). Afterward, we analyzed the correlations between 3 S-MMR score, the steps of the cancer immunity cycle (Fig. 7C, **left**), as well as the enrichment scores of 28 published immune cell signatures [50] (Fig. 7C, **right**). Subsequently, we determined the infiltration level of TIICs by using six independent immune predication algorithms (TIMER, CIBERSORT-ABS, quanTIseq, xCell, MCP-counter, TIP) (Fig. 7D). In line with aforementioned results, the majority algorithms demonstrated a negative correlation between the 3 S-MMR score and the infiltration levels of CD8 + T cells, NK cells, Th1 cells, macrophages, and dendritic cells, suggesting low 3 S-MMR score patients might as “immune-hot” tumors while high 3 S-MMR score patients as “immune-cold” tumors. The histopathological section also verified that the low 3 S-MMR score group displayed upregulated infiltration of immune cells, once again indicating a strong negative correlation between 3 S-MMR score as well as immune infiltration (Fig. 7E). Gene expression of IMs varied across immune subtypes, perhaps indicative of the 3 S-MMR score role in shaping the TME (Fig. 7F).

### 3 S-MMR score’s ability to predict immunotherapy efficacy

**Fig. 8:**
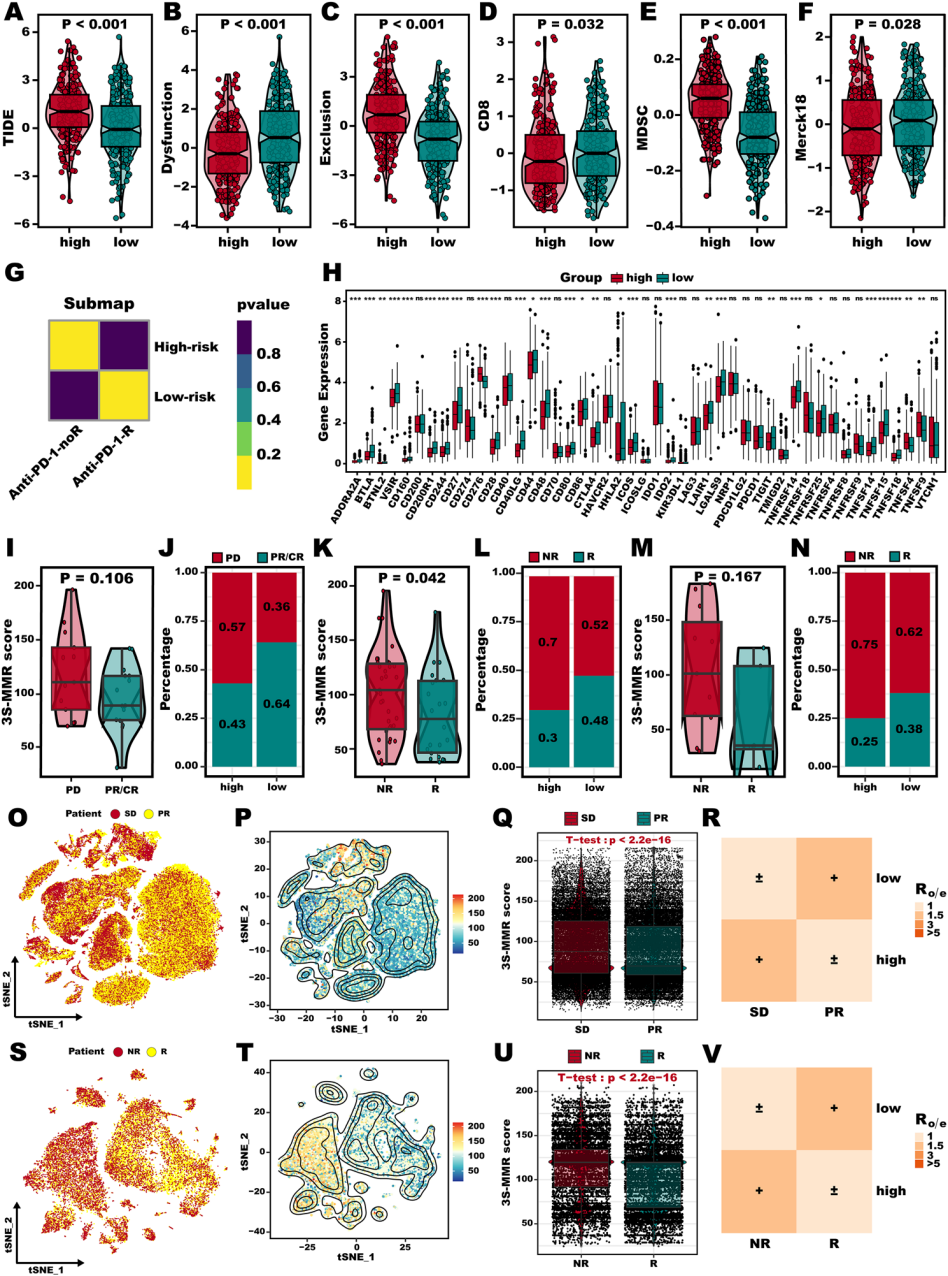
**3 S-MMR score’s ability to predict immunotherapy efficacy.** (**A-F**) Violin plot of TIDE (**A**), Dysfunction (**B**), Exclusion (**C**), CD8 (**D**) MDSC (**E**), and Merck18 (**F**) score. (**G**) The submap algorithm predict the response of high and low 3 S-MMR score groups to CTLA4 and PD-1 inhibitors. (**H**) Boxplot of relative expression levels at immune checkpoints profiles between the high and low 3 S-MMR score groups patients. (**I-N**) Differences in 3 S-MMR score between immunotherapy responders and non-responders in the GSE126044 (**I-J**), GSE35640 (**K-L**), and GSE78220 (**M-N**) cohorts. (**O-P**) T-SNE reduction maps the distribution of cells from SD and PR patients (**O**), and the distribution of 3 S-MMR score (**P**) in the GSE207422 dataset. (**Q**) Violin plot of 3 S-MMR score between SD and PR patients in the GSE207422 dataset. (**R**) Tissue preference of high and low 3 S-MMR groups estimate by *R*_o/e_ in the GSE207422 dataset. (**S-T**) T-SNE reduction maps the distribution of cells from SD and PR patients (**S**), and the distribution of 3 S-MMR score (**T**) in the GSE145281 dataset. (**U**) Violin plot of 3 S-MMR score between SD and PR patients in the GSE145281 dataset. (**V**) Tissue preference of high and low 3 S-MMR groups estimate by *R*_o/e_ in the GSE145281 dataset. Abbreviation: *P < 0.05; **P < 0.01; *** *P* < 0.001

The above results suggest that LUAD patients exhibiting a lower 3 S-MMR score might derive greater benefit from immunotherapy, given their tendency to have “immune-hot” tumors. Afterward, using the TIDE web tool, we found the TIDE (Fig. 8A), Exclusion (Fig. 8B), MDSC (Fig. 8E) scores was significantly elevated in the high 3 S-MMR score group, while Dysfunction (Fig. 8B), CD8 (Fig. 8D), and Merck18 (Fig. 8F) scores was significantly elevated in the low 3 S-MMR score group, which confirmed that patients with lower 3 S benefit more from immunotherapy. Submap algorithms further confirmed this conclusion (Fig. 8G). Acknowledging the profound effects of atypical expression and functionality of immune checkpoint molecules in tumor immunotherapy, we conducted an evaluation of immune checkpoints (ICs) expression within distinct 3 S-MMR score groups. Interestingly, almost all ICs exhibited increased expression in the group with low 3 S-MMR scores (Fig. 8H). Subsequently, we confirmed the effectiveness across various immunotherapy treatment cohorts. The 3 S-MMR score was lower in the CR/PR group than in the SD/PD group in the GSE78220 (Fig. 8I, P **= 0.106**), GSE35640 (Fig. 8K, P **= 0.042**), and GSE126044 cohorts (Fig. 8M, P **= 0.167**). We observed that 64% of patients in the CR/PR group had a low 3 S-MMR score in the GSE78220 (Fig. 8J), 48% of patients in the CR/PR group had a low 3 S-MMR score in the GSE35640 (Fig. 8L), and 38% of patients in the CR/PR group had a low 3 S-MMR score in the GSE126044 (Fig. 8N). These results were additionally validated through two scRNA-seq datasets that included data on immunotherapy. For patients responding to immunotherapy therapy, their cells had a significantly lower 3 S-MMR score than non-responders’ cells both in GSE207422 (Fig. 8O and R), and GSE145281 cohorts (Fig. 8S and V). Considering the aforementioned analysis, it can be hypothesized that immunotherapy might yield better outcomes in cancer patients who have a lower 3 S-MMR score.

**In silico identification of targets and drugs for high 3 S-MMR score patients.**

**Fig. 9:**
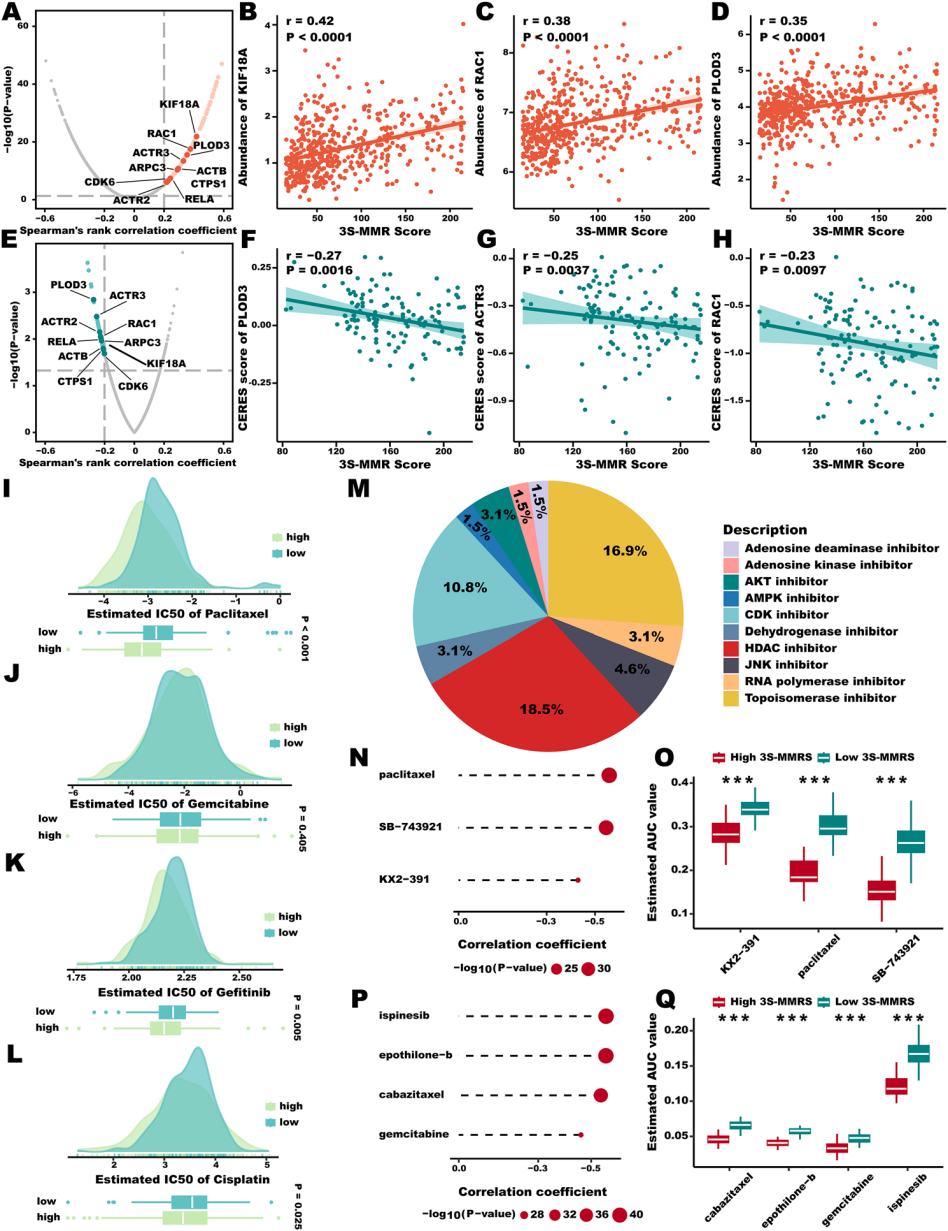
**In silico identification of targets and drugs for high 3 S-MMR score patients.** (**A-D**) Volcano plot (**A**) and scatter plots (**B-D**) of the correlation coefficients derived from Spearman’s rank correlation analysis between 3 S-MMR score and druggable mRNA expression in the TCGA-LUAD cohort. Red dots indicate the significant positive correlations (*P* < 0.05, and Spearman’s *r* > 0.2). (**E-H**) Volcano plot (**E**) and scatter plots (**F-H**) of the correlations and significance between 3 S-MMR score and CERES score of drug targets. Green dots indicate the significant negative correlations (*P* < 0.05, and Spearman’s *r* < -0.2). (**I-L**) The comparison of IC50 values between high and low 3 S-MMR score groups of Paclitaxel (**I**), Gemcitabine (**J**), Gefitinib (**K**), and Cisplatin (**L**). (**M**) The composition of chemical compounds selected by CMap analysis. Only the top 10 drug categories are displayed. (**N, P**) The result of Spearman’s correlation analysis of CTRP-derived compounds (**N**) and PRISM-derived compounds (**P**). (**O, Q**) The results of differential drug response analysis of CTRP-derived compounds (**O**) and PRISM-derived compounds (**Q**), the lower values on the y-axis of boxplots imply greater drug sensitivity. Abbreviation: *P < 0.05; **P < 0.01; *** *P* < 0.001

In order to screen potential targets for high 3 S-MMR score patients, we collected the target information of 6152 compounds and 2249 druggable targets (**Supplementary Table 9**) from previous study [39, 51] and applied two-step analysis to find candidate targets. To begin, we executed a Spearman’s correlation analysis to explore the association between the 3 S-MMR score and the expression levels of potential drug targets in the TCGA-LUAD cohort. From this, we identified a shared group of genes positively correlated with the score, designating these as related targets for the 3 S-MMR score (Fig. 9A and D, **Supplementary Table 10**, *n* = 269, ***r*** **> 0.2**, ***p*** **< 0.05**). Subsequently, by performing a Spearman’s correlation analysis between the CERES score and the 3 S-MMR score using lung cancer cell lines, we proceeded to identify 54 targets dependent on poor prognosis (Fig. 9E and H, **Supplementary Table 11**, *n* = 54, ***r*< -0.2**, ***p*** **< 0.05**). In total of ten genes, including CDK6, RELA, CTPS1, PLOD3, KIF18A, ACTR2, ACTR3, ARPC3, ACTB, and RAC1 were identified by both analyses above, suggesting that targeting these genes for inhibition in patients with high 3 S-MMR scores might result in enhanced treatment effectiveness. Besides, the IC50 values of target therapy drugs including paclitaxel (Fig. 9I), gemcitabine (Fig. 9J), gefitinib (Fig. 9K), and cisplatin (Fig. 9L) were lower in the high 3 S-MMR score groups, suggested that high 3 S-MMR score patients might more likely benefit from targeted therapy. Subsequently, we undertook CMap analysis to deduce potentially efficacious chemical compounds. For this purpose, we executed a differential gene expression analysis contrasting the high and low 3 S-MMR score groups. We then utilized the top 150 most upregulated as well as 150 most downregulated genes as the signature for the 3 S-MMR score to determine the CMap score for chemical compounds. By employing this method, we pinpointed a total of 65 compounds, each with a CMap score below − 95 and the capability to reverse the 3 S-MMR score signature (**Supplementary Table 12**). Of 65 compounds, 18.5% and 16.9% belong to HDAC inhibitors and topoisomerase inhibitors, respectively (Fig. 9M). Afterward, we applied Yang.et al’s protocol [39] to identify potentially sensitive drugs for the high 3 S-MMR score group, and finally generated three CTRP-derived drugs (KX2 − 391, paclitaxel, SB − 743,921) and four PRISM-derived drugs (cabazitaxel, epothilone − b, gemcitabine, ispinesib). The estimate AUC values of these drugs were not only statistically negatively correlated with 3 S-MMR scores, but also significantly lower in the high 3 S-MMR score groups (Fig. 9N and Q).

### Dissecting the malignant cells with 3 S-MMR score

**Fig. 10:**
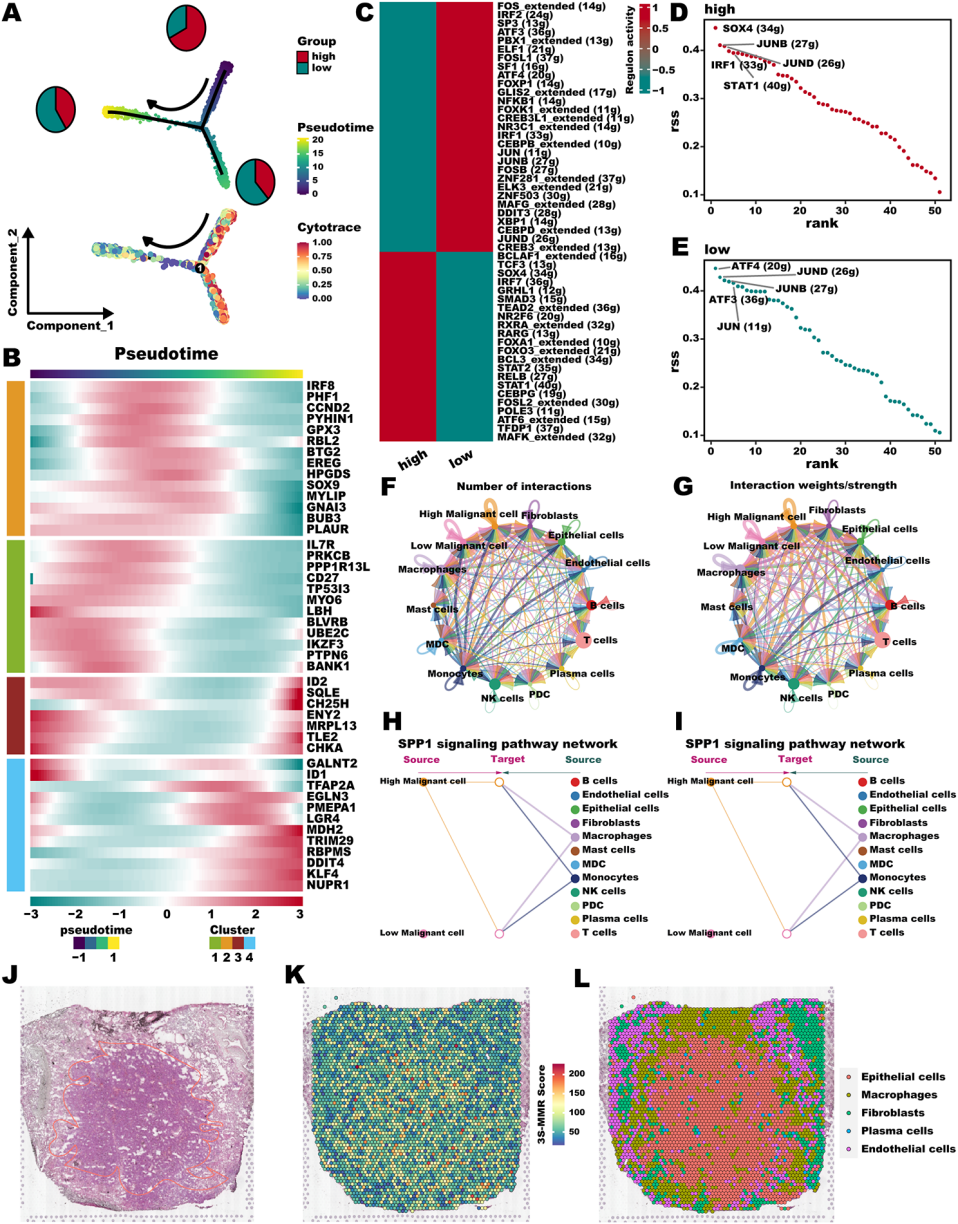
**Dissecting the malignant cells with high 3 S-MMR score.** (**A**) The development trajectory of malignant cells inferred by Monocle2. Malignant cells with high 3 S-MMR scores most located in the roots of differentiation, and the malignant cells with low 3 S-MMR scores mainly located in the middle and end-point state. (**B**) Heatmap of the 3 S-MMR score-related genes in malignant cells along the pseudo-time. (**C**) Heatmap showing the different TFs activation between high and low 3 S-MMR score malignant cells. (**D, E**) Top activities of TFs between high (**D**) and low 3 S-MMR (**E**) score of malignant cells. RSS indicates Regulon Specificity Score. (**F, G**) Cellchat analysis of all cell types. Both interaction numbers and interaction strengths were showed. (**H, I**) Hierarchical plot showing the inferred intercellular communication network for SPP1 signaling pathway. (**J**) HE staining showing histologically distinct regions of stRNA samples. yellow: cancer region. (**K**) The spatial plot of 3 S-MMR score intensity. (**L**) The distribution of different cell types in the spatial map was identified by the algorithm of RCTD.

Considering the presence of cells at various developmental stages within tumor tissue, we investigated the association between the 3 S-MMR score as well as the developmental trajectory of malignant cells using pseudo-time analysis. The trace plot for malignant cells, depicting fluctuations in the 3 S-MMR score over pseudo-time, exhibited three distinct branches (Fig. 10A, **upper panel**). We further applied CytoTRACE analysis by integrating Monocle2 to predict the origins of malignant cells (Fig. 10A, **lower panel**). Malignant cells with high 3 S-MMR scores were mainly located near the root node of the branch tree, indicated that malignant cells with high 3 S-MMR scores may be more characteristics of cancer stem cells. When we delineated the distribution of the 3 S-MMR score-related gene list (detach gene-pair), we noticed that they are expressed differently in various stages of malignant cells, which further confirms that 3 S-MMR score has the potential to regulate the differentiation and progression of malignant cells (Fig. 10B). In light of the fact that different cell types are distinguished by the outcomes of intricate and coordinated interactions involving transcription factors (TFs) and their associated target genes, we delved into the interconnectedness among transcription factors within malignant cells using SCENIC analysis. We found that malignant cells with high as well as low 3 S-MMR scores have completely different activation phenomena of transcription factors (Fig. 10C). Top 5 TFs in malignant cells with high 3 S-MMR scores included SOX4, IRF1, JUNB, JUND, and STAT1 (Fig. 10D), while ATF4, ATF3, and JUN was extended in malignant cells with low 3 S-MMR score (Fig. 10E). Subsequently, to investigate the differences in intercellular communication between high and low 3 S-MMR score malignant cells, we analyzed the expression of receptor and ligand. Figure 10F and G illustrates the communication between all cells, and we found that malignant cells with high 3 S-MMR scores was able to send signals more effectively than low 3 S-MMR scores malignant cells. Secreted phosphoprotein 1 (SPP1), an extracellular glycoprotein with phosphorylated residues, exhibits a close association with various aspects of tumor biology, notably proliferation, migration, and invasion, particularly in the context of LUAD [52]. Analysis of the network centrality showed that malignant cells with high 3 S-MMR scores plan an important role in SPP1 signaling pathway (Fig. 10H and I). We further applied 3 S-MMR framework to the spatial architecture of LUAD. Afterward, based on HE staining, we labeled the cancerous area (Fig. 10J). It’s worth highlighting that malignant cells characterized by higher 3 S-MMR scores are primarily situated at the central core of the LUAD tumor (Fig. 10K). Furthermore, we utilized the robust cell type decomposition (RCTD) algorithm [53] to extrapolate the identified cell types from single-cell data to the spatial dataset, allowing us to infer the predominant cell types present at each spatial location (Fig. 10L). This outcome further strength the conclusion that cells exhibiting higher 3 S-MMR scores are primarily situated within the tumor epithelial cell region.

## Pan-cancer analysis

**Fig. 11:**
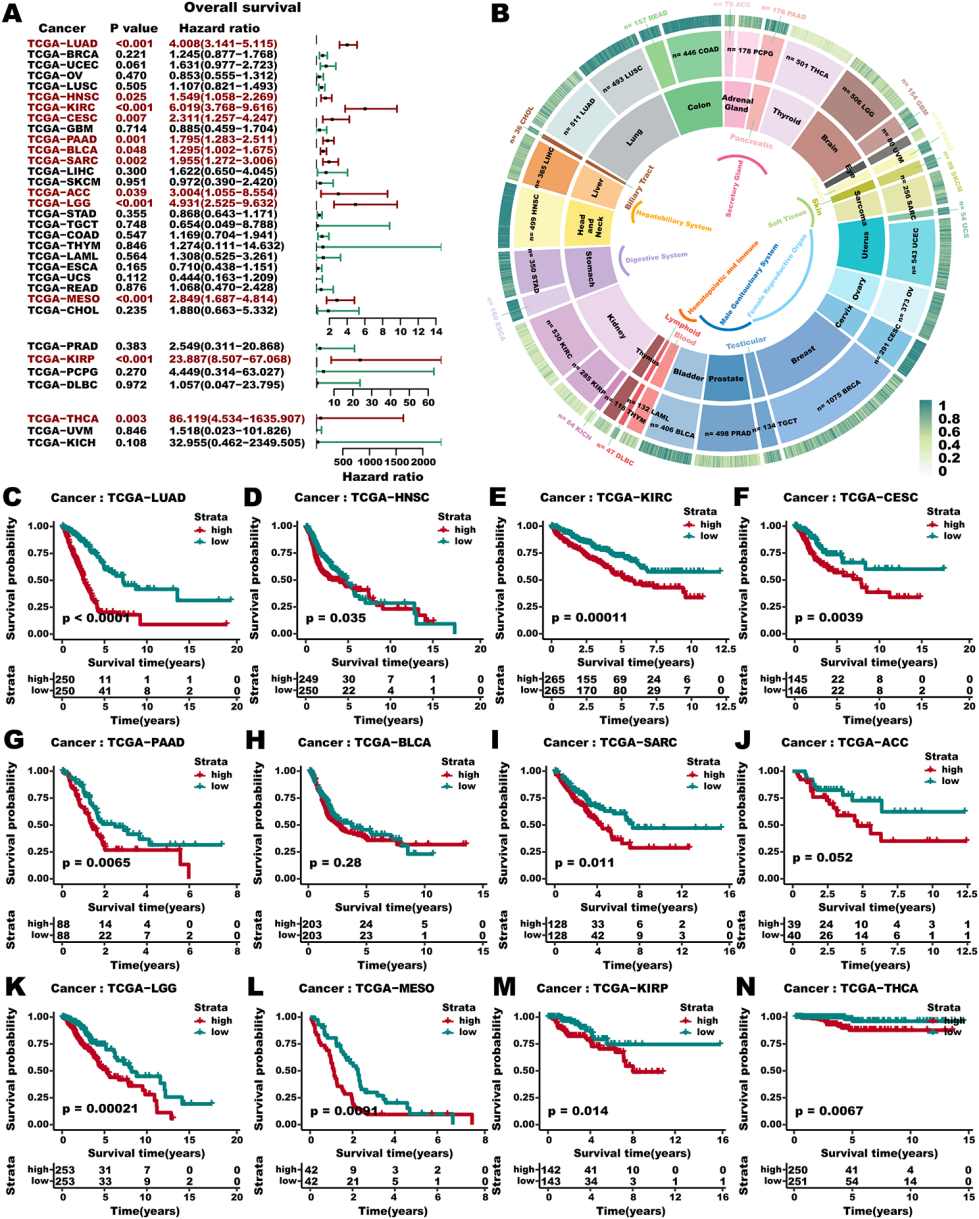
**Pan-cancer analysis.** (**A)** Cox regression analysis of 3 S-MMR score across 33 cancer types. Red color indicates *P <* 0.05 significant results. (**B**) Average 3 S-MMR score in individual cancer types. Tissue types, cancer types and average 3 S-MMR score are shown from the inner circle to the outer circle. (**C-N**) Kaplan–Meier survival curves of 3 S-MMR score in 12 types of cancers are significant (Log-rank test)

Drawing from previous studies indicating that pan-cancer-related pathways also exhibited similar, and common activity profiles to the LUAD pathway among the prognostic groups [54] as well as considering metabolic reprogramming as a necessity for tumor progression and metastasis [55], we subsequently delved into the broader potential utility of the 3 S-MMR score in various other cancer types. We conducted a pan-cancer analysis using expression and survival data from 33 cancer types (*n* = 10,110) in the TCGA dataset, which encompassed two hematological cancers and 31 solid tumors. Univariate Cox regression outcomes showed that the 3 S-MMR score was a unfavorable prognostic factor across all cancer types (all HR > 1), significantly in LUAD, HNSC, KIRC, CESC, PAAD, BLCA, SARC, ACC, LGG, MESO, KIRP, and THCA (Fig. 11A). Tissue-specific senescence levels across cancer types were further revealed (Fig. 11B). Additionally, the 3 S-MMR score can distinguish survival states in most cancers (Fig. 11C and N), among which the low 3 S-MMR score group exhibited longer survival time. These findings validate the viability of employing the 3 S-MMR score in these types of cancers.

## Discussion

Cancer cells must respond swiftly to both internal and external triggers in order to sustain their rapid proliferation and survival in adverse conditions characterized by low oxygen levels, nutritional scarcity, and potential exposure to chemotherapy drugs [56]. An essential strategy involves the reprogramming of cellular metabolism, thereby influencing both intra- and extra-cellular metabolites, with the potential to significantly impact gene expression, cellular differentiation, and the TME [57, 58]. Metabolic reprogramming, notably in the realm of energy metabolism, has been universally recognized as a hallmark and prevalent characteristic of tumors for a significant duration [2]. Recent investigations into metabolic reprogramming have unveiled promising potential targets for cancer treatment [59, 60], underscoring the substantial need for comprehensive research on metabolic heterogeneity and the associated metabolic mechanisms. Predominantly reliant on bulk RNA-seq data, prior transcriptomics investigations in LUAD predominantly concentrated on the identification of prognostic genes and the prediction of clinical outcomes. However, they often overlooked the intricate landscape of intercellular heterogeneity. Simultaneously, scRNA-seq studies on LUAD mainly focused on functions of various cell component [61], discovery of new cell subsets [62], and investigation of intercellular heterogeneity [15], but were lack of the analyses between specific tumor cell subsets and patient’s prognosis for the limitation of small sample size. Furthermore, the majority of prior models were constrained by biases arising from the reliance on absolute gene expression values. These models predominantly focused on singular cancer-related pathways and often employed inappropriate modeling methodologies. Such limitations substantially impeded the robustness and accuracy of their predictions, thereby hindering their practical applicability.

In this study, we first leverage scRNA-seq profiles to study the metabolic heterogeneity in primary as well as metastatic samples form LUAD patients. Based on the cell level and complexity ecosystem, we defined a new gene set which related to LUAD progression and metastatic, named “MMR”. Through the integration of GA and ensemble learning methodologies, leveraging the relative expression order of gene pairs within samples, we eliminated the necessity for data normalization, enhancing reliability and generalizability, and further proposing 3 S-MMR score, with it exhibiting a powerful ability in survival prediction of LUAD patients. To the best of our knowledge, this is the first prognostic model in LUAD based on ensemble learning that simultaneously considers four key points involving malignant and metabolism reprogramming, gene-pair, double training sets as well as GA algorithm. Utilizing nine independent LUAD cohorts, we illustrated that the 3 S-MMR score exhibits superior robustness and accuracy compared to existing clinical indicators. Additionally, we provided a web tool for the 3 S-MMR score-based nomogram, facilitating the user-friendly application of the risk score for LUAD patients.

It’s important to highlight that the 3 S-MMR scoring framework we formulated tackles batch discrepancies among diverse datasets, maintaining the integrity of the initial data distribution. This is advantageous for the enhancement of subsequent ensemble learning models. Regardless of the transcriptome platform employed and the data formats utilized, such as FPKM, TPM, and RPKM, the gene-pairing approach simplifies both the comparative analysis and the amalgamation of data. Moreover, it’s important to mention that by employing double training sets, we effectively minimized overfitting, thereby enhancing the precision of the 3 S-MMR score in the test sets. Genetic algorithms automated the selection of fundamental learners in the ensemble learning model, further contributing to enhanced accuracy. Certainly, it is pertinent to recognize that, as previously discussed, our framework, grounded in the fundamental schema of cancer cell metabolic reprogramming, also extensively addresses the issue of heterogeneity in transcriptomic platforms, clinical attributes, and genetic profiles of these LUAD cohorts. This was further corroborated in subsequent pan-cancer analyses, indicating that the 3 S-MMRscore, built on the universal survival foundation of cancer cells and derived from metabolic reprogramming, acts as a risk factor across various other cancers (Fig. 11A, HR > 1).

Prior research indicates that alterations in cancer cell metabolism are partly attributable to the recruitment of numerous inflammatory and immune cells [63]. Following this, an increasing number of scientists have discovered that aberrant metabolites or intermediates of cancer metabolism play a crucial role in controlling the proliferation, differentiation, activation, and functionality of immune cells. Recent research has shed light on a previously unappreciated intricate link between the immune system and various metabolic processes, leading to the emergence of a new field termed immunometabolism [58, 64]. In our study, we found that the 3 S-MMR scoring system can significantly distinguish different immune infiltration patterns, indicating its potential to participate in immune remodeling, which is likely to be regulated through metabolic reprogramming.

In this study, we found that 3 S-MMR score was negatively correlated with the activities of several steps of the cancer immunity cycle. For instance, the activity of T cell recruitment was significantly upregulated in the low 3 S-MMR score group. Consequently, the infiltration levels of several effector TIICs, such as CD8 + T cells, CD4 + T cells, NK cells, macrophages, TH1 cells, as well as dendritic cells, were also significantly upregulated. These findings are verifiable across various algorithms. An essential feature of an inflamed TME includes the escalated activation of inhibitory immune checkpoints, influenced by the initial influx of TIICs [65]. These immune checkpoints dampen pre-established cancer immunity to prevent an overactive immune response, yet they also contribute to immune escape. ICB therapies targeting these checkpoints have yielded significant survival advantages in advanced LUAD cases [66]. Here, the high 3 S-MMR score group exhibited a notable decrease in the expression of inhibitory immune checkpoints, possibly due to the reduced activity of pre-existing TIICs. This implies that LUAD cases with a high 3 S-MMR score may exhibit reduced sensitivity to ICB treatment. Besides, TIDE, submap, and independent bulk immunotherapy cohorts confirmed this conclusion. It’s noteworthy that, in our analysis of several single-cell cohorts undergoing immunotherapy, we observed a marked trend where cells exhibiting high 3 S-MMR scores predominantly originated from patients who did not respond to the treatment. On the contrary, the significant negative correlation between 3 S-MMR score and targeted therapy drugs indicated that patients with high 3 S-MMR scores might more likely benefit from targeted therapy. The use of in silico identification for drug and target screening has been shown in many literatures to be a very promising strategy for targeting specific patient populations [67–70]. For instance, Faheem Ahmed et al. introduced an integrative drug repurposing framework that relies on a systems biology-enabled network medicine platform to efficiently identify suitable repurposable drugs and drug combinations for targeting HPV-associated cervical cancer [71]. In our study, we identified 10 potential therapeutic targets (e.g., PLOD3, ACTR3, and RAC1), along with three CTRP-derived and four PRISM-derived therapeutic agents, for patients with high 3 S-MMR scores. In the future, more clinical trials are required to confirmed broad prospects of these target genes and therapeutic agents.

We also attach great importance to the biological significance behind this prognosis and the huge differences in immune infiltration patterns. When we applied the 3 S-MMR score framework to the scRNA-seq level, we found that there were large differences in malignant cells with high as well as low 3 S-MMR scores. Regarding the analysis of cellular communication, it was noted that, generally, malignant cells with elevated 3 S-MMR scores exhibited pronounced interactions with immune cells, which play a crucial role in tumor progression and metastasis. Considering the presence of cells at varying developmental stages within tumor tissue, we investigated the association between the 3 S-MMR score and the developmental trajectory of malignant cells using pseudo-time analysis. It appeared that malignant cells with a high 3 S-MMR score were more indicative of cancer stem cell characteristics, whereas cells with a low 3 S-MMR score tended to be closer to the differentiated end-stage. Similarly, we found that 3 S score-related genes differ greatly at different stages of malignant cell progression. We have reason to believe that the 3 S-MMR score framework has a higher ability to regulate malignant cell differentiation. Additionally, by incorporating the 3 S-MMR score framework into the LUAD spatial transcriptome through deconvolution, we observed that the central regions of LUAD tumors typically exhibit relatively higher 3 S-MMR scores.

Although we confirmed the accuracy of the 3 S-MMR process results, it’s important to acknowledge certain limitations of this method, including the possible adverse impact on model efficacy due to the data partitioning approach. Moreover, while LUAD patients provide ample data for implementing the 3 S-MMR score, other cancers might lack enough scRNA-seq and bulkRNA-seq samples for constructing a 3 S-MMR pipeline, potentially restricting the applicability of the 3 S-MMR score.

### Electronic supplementary material

Below is the link to the electronic supplementary material.

Supplementary Material 1

Supplementary Material 2

## Data Availability

All data used in this work can be acquired from the TCGA Research Network portal (https://portal.gdc.cancer.gov/) and GEO (https://www.ncbi.nlm.nih.gov/geo/).

## References

[1] Hanahan D (2022). Hallmarks of Cancer: New dimensions. Cancer Discov.

[2] Ge T (2022). Crosstalk between metabolic reprogramming and epigenetics in cancer: updates on mechanisms and therapeutic opportunities. Cancer Commun (Lond).

[3] Zanotelli MR, Zhang J, Reinhart-King CA (2021). Mechanoresponsive Metabolism cancer cell Migration Metastasis Cell Metab.

[4] Tan Y (2022). Metabolic reprogramming from glycolysis to fatty acid uptake and beta-oxidation in platinum-resistant cancer cells. Nat Commun.

[5] Koundouros N, Poulogiannis G (2020). Reprogramming of fatty acid metabolism in cancer. Br J Cancer.

[6] Lopez Krol A (2022). Lactate induces metabolic and epigenetic reprogramming of pro-inflammatory Th17 cells. EMBO Rep.

[7] Pavlova NN, Zhu J, Thompson CB (2022). The hallmarks of cancer metabolism: still emerging. Cell Metab.

[8] Qian Y (2023). MCT4-dependent lactate secretion suppresses antitumor immunity in LKB1-deficient lung adenocarcinoma. Cancer Cell.

[9] Li Y (2023). PINK1-Mediated Mitophagy promotes oxidative phosphorylation and Redox Homeostasis to Induce Drug-Tolerant Persister Cancer cells. Cancer Res.

[10] Zheng X (2023). Single-cell analyses implicate ascites in remodeling the ecosystems of primary and metastatic tumors in ovarian cancer. Nat Cancer.

[11] Kaur I (2021). An Integrated Approach for Cancer Survival Prediction using Data Mining techniques. Comput Intell Neurosci.

[12] Zhu S et al. The genetic algorithm-aided three-stage ensemble learning method identified a robust survival risk score in patients with glioma. Brief Bioinform, 2022. 23(5). 10.1093/bib/bbac344 36088543

[13] Swanson K (2023). From patterns to patients: advances in clinical machine learning for cancer diagnosis, prognosis, and treatment. Cell.

[14] Kong W et al. *Adaptive best subset selection algorithm and genetic algorithm aided ensemble learning method identified a robust severity score of COVID-19 patients* 2023. 2(3): p. e126. 10.1002/imt2.126 PMC10989835 38867930

[15] Kim N (2020). Single-cell RNA sequencing demonstrates the molecular and cellular reprogramming of metastatic lung adenocarcinoma. Nat Commun.

[16] Yang C (2020). Metabolism-associated molecular classification of hepatocellular carcinoma. Mol Oncol.

[17] Butler A (2018). Integrating single-cell transcriptomic data across different conditions, technologies, and species. Nat Biotechnol.

[18] Korsunsky I (2019). Fast, sensitive and accurate integration of single-cell data with Harmony. Nat Methods.

[19] Tirosh I (2016). Dissecting the multicellular ecosystem of metastatic melanoma by single-cell RNA-seq. Science.

[20] Zhao S (2023). Combining single-cell sequencing and spatial transcriptome sequencing to identify exosome-related features of glioblastoma and constructing a prognostic model to identify BARD1 as a potential therapeutic target for GBM patients. Front Immunol.

[21] Andreatta M, Carmona SJ (2021). UCell: robust and scalable single-cell gene signature scoring. Comput Struct Biotechnol J.

[22] Clynick B et al. Biomarker signatures for progressive idiopathic pulmonary fibrosis. Eur Respir J, 2022. 59(3). 10.1183/13993003.01181-2021 34675050

[23] Liu Z (2022). Machine learning-based integration develops an immune-derived lncRNA signature for improving outcomes in colorectal cancer. Nat Commun.

[24] Sparapani R (2020). Nonparametric competing risks analysis using bayesian additive regression trees. Stat Methods Med Res.

[25] Gonzalez-Angulo AM (2013). Functional proteomics characterization of residual breast cancer after neoadjuvant systemic chemotherapy. Ann Oncol.

[26] Natekin A, Knoll A (2013). Gradient boosting machines, a tutorial. Front Neurorobot.

[27] Longato E, Vettoretti M, Di Camillo B (2020). A practical perspective on the concordance index for the evaluation and selection of prognostic time-to-event models. J Biomed Inf.

[28] Reader AJ, Ellis S (2020). Bootstrap-optimised regularised Image Reconstruction for Emission Tomography. IEEE Trans Med Imaging.

[29] Zhang Z (2016). Statistical description for survival data. Ann Transl Med.

[30] Lynch CM (2017). Prediction of lung cancer patient survival via supervised machine learning classification techniques. Int J Med Inf.

[31] Feng X, Zhao J, Kita E (2021). Genetic algorithm-based optimization of deep neural network ensemble. Rev Socionetwork Strategies.

[32] Jia L et al. Development of interactive biological web applications with R/Shiny. Brief Bioinform, 2022. 23(1). 10.1093/bib/bbab415 34642739

[33] Jin S (2021). Inference and analysis of cell-cell communication using CellChat. Nat Commun.

[34] Aibar S (2017). SCENIC: single-cell regulatory network inference and clustering. Nat Methods.

[35] Qiu X (2017). Single-cell mRNA quantification and differential analysis with Census. Nat Methods.

[36] Cheng S (2021). A pan-cancer single-cell transcriptional atlas of tumor infiltrating myeloid cells. Cell.

[37] Gulati GS (2020). Single-cell transcriptional diversity is a hallmark of developmental potential. Science.

[38] Ghandi M (2019). Next-generation characterization of the Cancer Cell Line Encyclopedia. Nature.

[39] Yang C et al. Prognosis and personalized treatment prediction in TP53-mutant hepatocellular carcinoma: an in silico strategy towards precision oncology. Brief Bioinform, 2021. 22(3). 10.1093/bib/bbaa164 32789496

[40] Subramanian A (2017). A Next Generation Connectivity Map: L1000 platform and the first 1,000,000 profiles. Cell.

[41] Ahmed F (2022). Drug repurposing in psoriasis, performed by reversal of disease-associated gene expression profiles. Comput Struct Biotechnol J.

[42] Maeser D, Gruener RF, Huang RS. oncoPredict: an R package for predicting in vivo or cancer patient drug response and biomarkers from cell line screening data. Brief Bioinform, 2021. 22(6). 10.1093/bib/bbab260 PMC8574972 34260682

[43] Zhang L (2018). Lineage tracking reveals dynamic relationships of T cells in colorectal cancer. Nature.

[44] Wu Y (2022). Spatiotemporal Immune Landscape of Colorectal Cancer Liver Metastasis at single-cell level. Cancer Discov.

[45] Xiao Z, Dai Z, Locasale JW (2019). Metabolic landscape of the tumor microenvironment at single cell resolution. Nat Commun.

[46] Leek JT (2012). The sva package for removing batch effects and other unwanted variation in high-throughput experiments. Bioinformatics.

[47] Fawcett C, Hoos HH (2016). Analysing differences between algorithm configurations through ablation. J Heuristics.

[48] Chen DS, Mellman I (2013). Oncology meets immunology: the cancer-immunity cycle. Immunity.

[49] Xu L (2018). TIP: a web server for resolving Tumor Immunophenotype profiling. Cancer Res.

[50] Charoentong P (2017). Pan-cancer immunogenomic analyses reveal genotype-immunophenotype relationships and predictors of response to checkpoint blockade. Cell Rep.

[51] Huang RH (2023). A machine learning framework develops a DNA replication stress model for predicting clinical outcomes and therapeutic vulnerability in primary prostate cancer. J Transl Med.

[52] Yi X (2022). SPP1 facilitates cell migration and invasion by targeting COL11A1 in lung adenocarcinoma. Cancer Cell Int.

[53] Cable DM (2022). Robust decomposition of cell type mixtures in spatial transcriptomics. Nat Biotechnol.

[54] Chen RJ (2022). Pan-cancer integrative histology-genomic analysis via multimodal deep learning. Cancer Cell.

[55] Yoshida GJ (2015). Metabolic reprogramming: the emerging concept and associated therapeutic strategies. J Exp Clin Cancer Res.

[56] Butler M, van der Meer LT, van Leeuwen FN (2021). Amino acid depletion therapies: starving Cancer cells to death. Trends Endocrinol Metab.

[57] Kroemer G, Pouyssegur J (2008). Tumor cell metabolism: cancer’s Achilles’ heel. Cancer Cell.

[58] Xia L (2021). The cancer metabolic reprogramming and immune response. Mol Cancer.

[59] Cheng C (2018). Lipid metabolism reprogramming and its potential targets in cancer. Cancer Commun (Lond).

[60] Ahmad F, Cherukuri MK, Choyke PL (2021). Metabolic reprogramming in prostate cancer. Br J Cancer.

[61] Lambrechts D (2018). Phenotype molding of stromal cells in the lung tumor microenvironment. Nat Med.

[62] Zhu J (2022). Delineating the dynamic evolution from preneoplasia to invasive lung adenocarcinoma by integrating single-cell RNA sequencing and spatial transcriptomics. Exp Mol Med.

[63] Biswas SK (2015). Metabolic reprogramming of Immune cells in Cancer Progression. Immunity.

[64] Artyomov MN, Van den Bossche J (2020). Immunometabolism Single-Cell Era Cell Metab.

[65] Spranger S (2013). Up-regulation of PD-L1, IDO, and T(regs) in the melanoma tumor microenvironment is driven by CD8(+) T cells. Sci Transl Med.

[66] Chi A et al. Classification of Non-Small Cell Lung Cancer’s Tumor Immune Micro-Environment and Strategies to Augment Its Response to Immune Checkpoint Blockade. Cancers (Basel), 2021. 13(12). 10.3390/cancers13122924 PMC8230820 34208113

[67] Ahmed F (2023). A systematic review of computational approaches to understand cancer biology for informed drug repurposing. J Biomed Inf.

[68] Ahmed F (2022). SperoPredictor: an Integrated Machine Learning and Molecular Docking-based drug Repurposing Framework with Use Case of COVID-19. Front Public Health.

[69] Ahmed F et al. Network-based drug repurposing identifies small molecule drugs as immune checkpoint inhibitors for endometrial cancer. Mol Divers, 2024. 10.1007/s11030-023-10784-7 38227161

[70] Ahmed F (2023). Drug repurposing for viral cancers: a paradigm of machine learning, deep learning, and virtual screening-based approaches. J Med Virol.

[71] Ahmed F (2023). Network-based drug repurposing for HPV-associated cervical cancer. Comput Struct Biotechnol J.

