# Supplementary material for: Architecting the metabolic reprogramming survival risk framework in LUAD through single-cell landscape analysis: three-stage ensemble learning with genetic algorithm optimization
Source: J Transl Med. 2024 Apr 15;22:353. doi: 10.1186/s12967-024-05138-2 (PMC11017668; doi:10.1186/s12967-024-05138-2)
Supplement: Supplementary file 1 — Supplementary Material 1 [file 12967_2024_5138_MOESM1_ESM.docx]

**Supplementary Figure 1:**


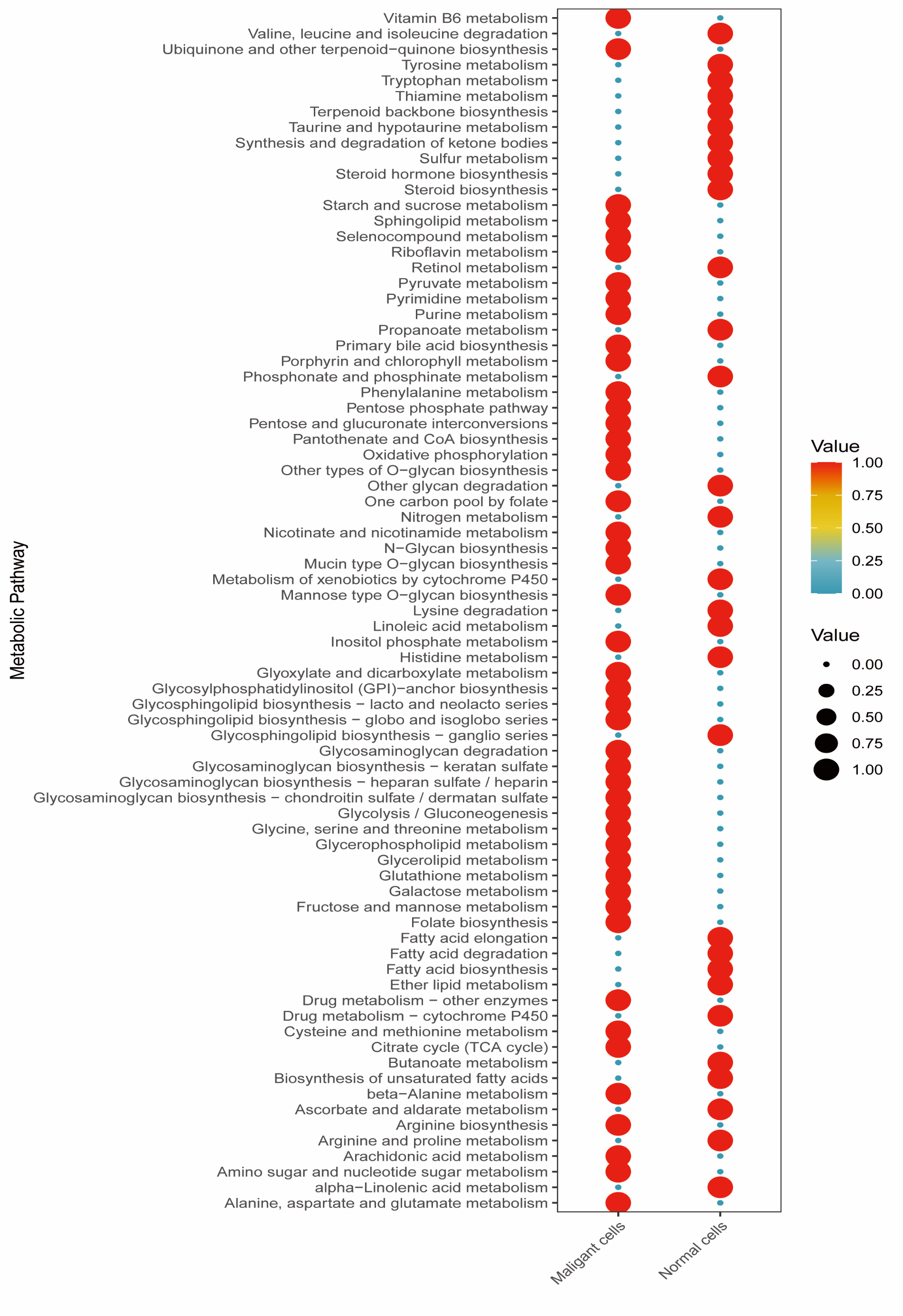

**Supplementary Figure 1: Dotplot showing directly metabolic characteristics between normal and malignant cells by using scMetabolism anaysis.**

**Supplementary Figure 2:**

**
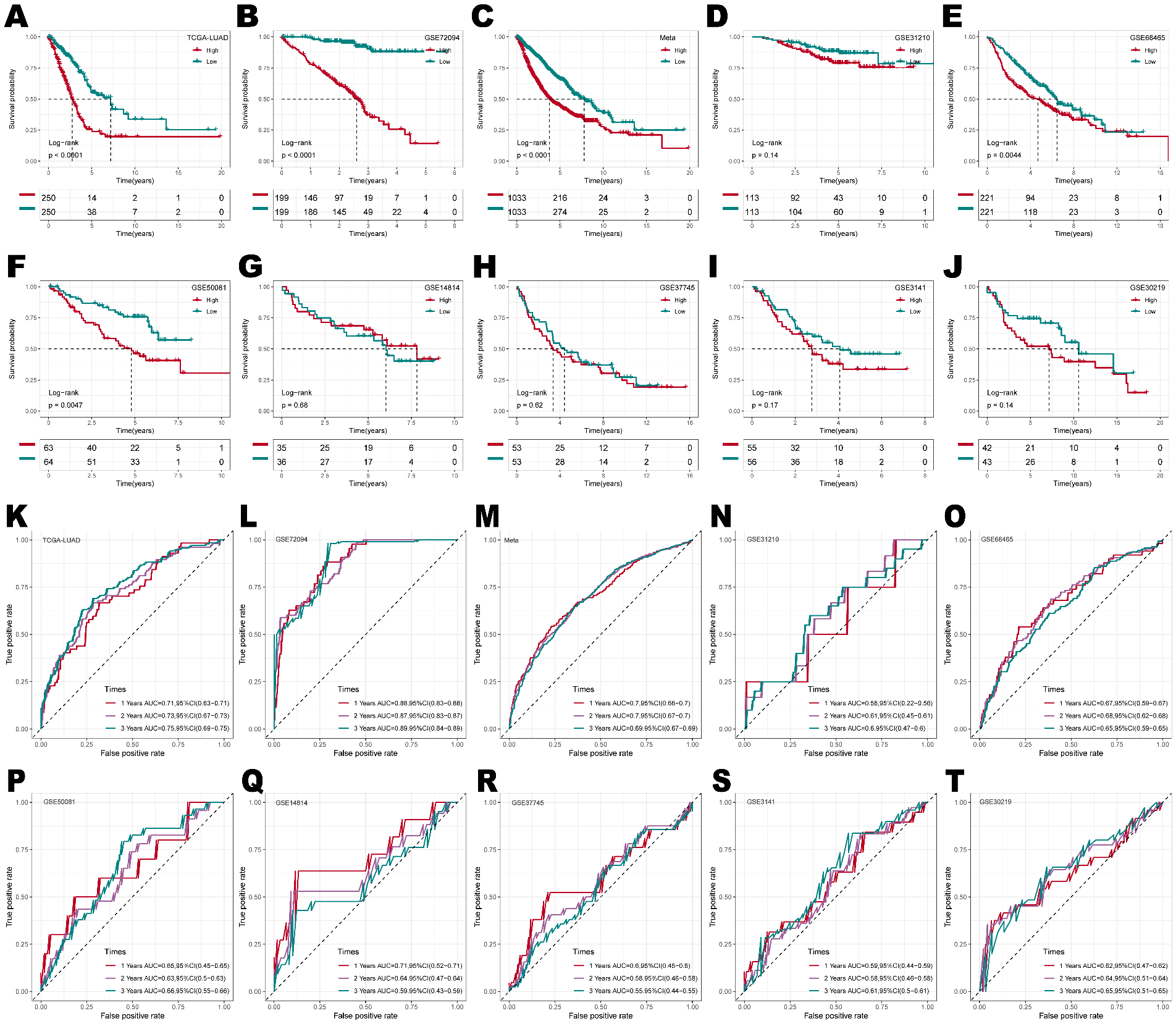
**

**Supplementary Figure 2: Evaluation of 3S-MMR score.** Kaplan-Meier survival curves of 3S-MMR score (without gene-pair method) in the training set 1 (TCGA-LUAD) (**A**), training set 2 (GSE72094) (**B**), meta (**C**), GSE31210 (**D**), GSE68465 (**E**), GSE50081 (**F**), GSE14814 (**G**), GSE37745 (**H**), GSE3141 (**I**), and GSE30219 (**J**) cohorts. ROC curves of 3S-MMR score in the training set 1 (TCGA-LUAD) (**K**), training set 2 (GSE72094) (**L**), meta (**M**), GSE31210 (**N**), GSE68465 (**O**), GSE50081 (**P**), GSE14814 (**Q**), GSE37745 (**R**), GSE3141 (**S**), and GSE30219 (**T**) cohorts.

**Supplementary Figure 3:**

**
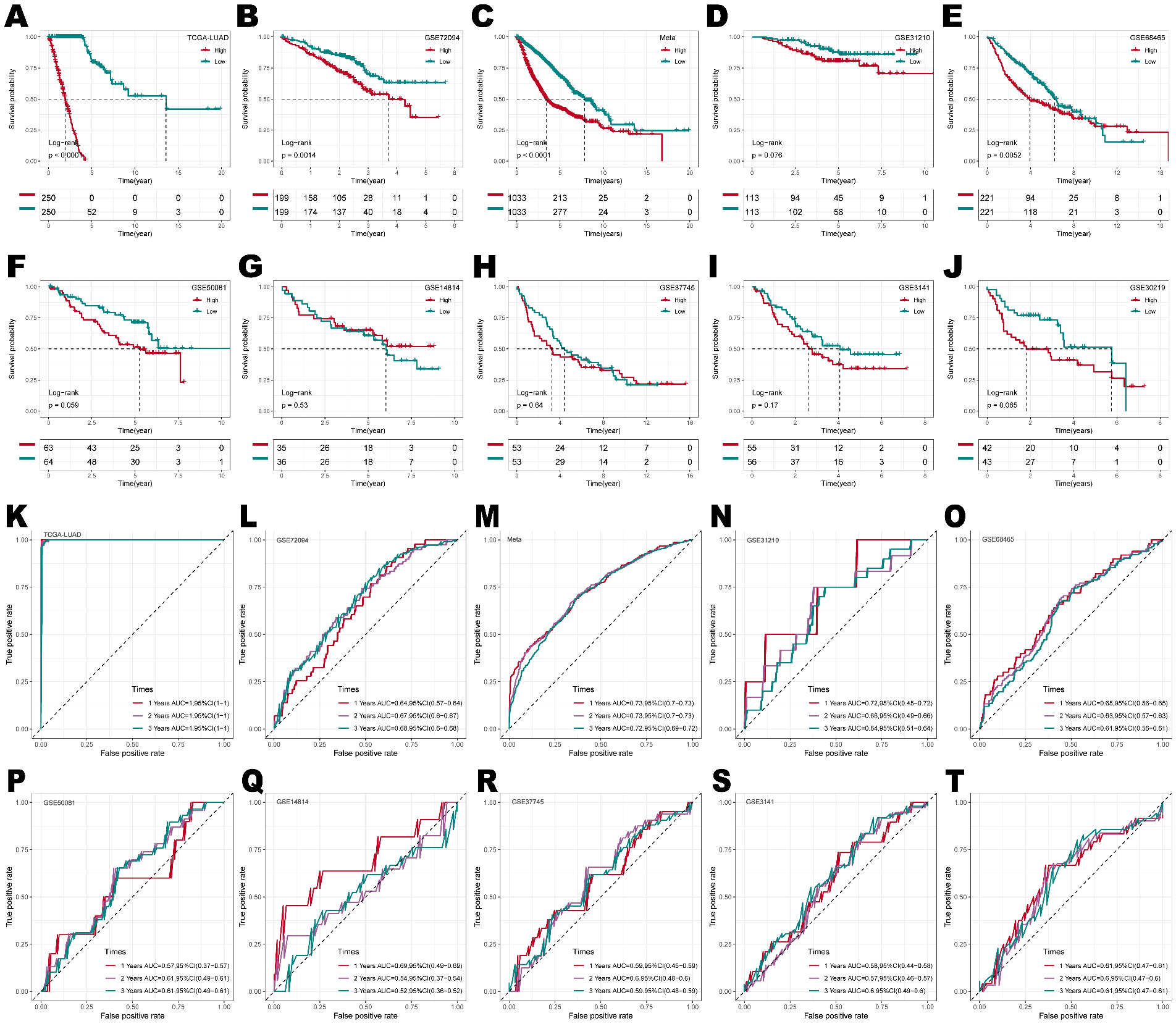
**

**Supplementary Figure 3: Evaluation of 3S-MMR score.** Kaplan-Meier survival curves of 3S-MMR score (without double training sets) in the training set (TCGA-LUAD) (**A**), GSE72094 (**B**), meta (**C**), GSE31210 (**D**), GSE68465 (**E**), GSE50081 (**F**), GSE14814 (**G**), GSE37745 (**H**), GSE3141 (**I**), and GSE30219 (**J**) cohorts. ROC curves of 3S-MMR score in the training set (TCGA-LUAD) (**K**), GSE72094 (**L**), meta (**M**), GSE31210 (**N**), GSE68465 (**O**), GSE50081 (**P**), GSE14814 (**Q**), GSE37745 (**R**), GSE3141 (**S**), and GSE30219 (**T**) cohorts.
